# Geometry‐derived statistical significance: A probabilistic framework for detecting true positive findings in MRI data

**DOI:** 10.1002/brb3.2865

**Published:** 2023-03-03

**Authors:** Ravi Bansal, Bradley S. Peterson

**Affiliations:** ^1^ Institute for the Developing Mind Children's Hospital Los Angeles California USA; ^2^ Department of Pediatrics and Psychiatry Keck School of Medicine at the University of Southern California Los Angeles California USA; ^3^ Department of Psychiatry Keck School of Medicine at the University of Southern California Los Angeles California USA

**Keywords:** arterial spin labeling, brain MRI, false discovery rate, false negatives, functional MRI, multiple comparisons

## Abstract

**Introduction:**

The false discovery rate (FDR) procedure does not incorporate the geometry of the random field and requires high statistical power at each voxel, a requirement not satisfied by the limited number of participants in imaging studies. Topological FDR, threshold free cluster enhancement (TFCE), and probabilistic TFCE improve statistical power by incorporating local geometry. However, topological FDR requires specifying a cluster defining threshold and TFCE requires specifying transformation weights.

**Methods:**

Geometry‐derived statistical significance (GDSS) procedure overcomes these limitations by combining voxelwise *p*‐values for the test statistic with the probabilities computed from the local geometry for the random field, thereby providing substantially greater statistical power than the procedures currently used to control for multiple comparisons. We use synthetic data and real‐world data to compare its performance against the performance of these other, previously developed procedures.

**Results:**

GDSS provided substantially greater statistical power relative to the comparator procedures, which was less variable to the number of participants. GDSS was more conservative than TFCE: that is, it rejected null hypotheses at voxels with much higher effect sizes than TFCE. Our experiments also showed that the Cohen's D effect size decreases as the number of participants increases. Therefore, sample size calculations from small studies may underestimate the participants required in larger studies. Our findings also suggest effect size maps should be presented along with *p*‐value maps for correct interpretation of findings.

**Conclusions:**

GDSS compared with the other procedures provides considerably greater statistical power for detecting true positives while limiting false positives, especially in small sized (<40 participants) imaging cohorts.

## 1 INTRODUCTION

Magnetic resonance imaging (MRI) provides exquisitely detailed measures of brain structure, function, and metabolism. The spatial resolution of MRI datasets has increased steadily while retaining excellent signal‐to‐noise ratios, due to the development of higher field strength MRI scanners, multi‐channel head coils, stronger and faster gradients, and parallel imaging techniques (Larkman et al., 2001; Nunes et al., 2006; Todd et al., 2016). A spatial resolution of 0.8mm^3^ yields data at 2.5 million voxels across the brain, almost double the number at a resolution of 1 mm^3^. Testing null hypotheses at each of these voxels poses a formidable multiple comparisons problem and risks reporting of large numbers of false positives in the findings. While true positive findings tend to form clusters, false positive findings tend to be scattered across the brain as clusters of few voxels. False positives cause poor reproducibility across studies and lead to incorrect conclusions about the brain processes under study. As a consequence, statistical procedures have been developed that help to reduce the rate of false positive findings in MR data analyses.

Early familywise error rate (FWER) procedures, including Bonferroni correction (Bonferroni, 1935, 1936), random field‐based procedures (Bansal et al., 2007; Friston & ebrary Inc., 2007; Friston et al., 1996, 1995), and cluster size approaches (Bansal & Peterson, 2018; Eklund et al., 2016; Friston et al., 1994) aimed to prevent any false positive findings. These procedures control for any false positive finding across all null hypotheses that are tested, and therefore, provide low statistical power to detect true positive findings, leading to high false negative rates, or type II errors. False negative findings interfere with forming correct conclusions about the processes under study every bit as much as false positive findings do. They also contribute to difficulties in replicating findings across studies. These limitations motivated the development of statistical procedures that more optimally balance between limiting false positive findings while simultaneously limiting false negatives.

False discovery rate (FDR) procedure (Benjamini, 2010; Benjamini & Hochberg, 1995a, 1995b) increases statistical power to detect true positives by prespecifying a fraction of false positives among all findings reported as being statistically significant. The FDR procedure estimates the threshold for statistical significance from the distribution of *p*‐values for the voxelwise statistical tests, and therefore can adapt to the amount of noise and signal in the data. Because FDR procedure is less conservative and provides greater statistical power than FWER, it increasingly is being used for assessing statistical significance for MRI *p*‐values. FDR procedure, however, can yield false negative findings in large clusters of voxels that are spatially contiguous if *p*‐values are not sufficiently small, and it may yield false positive findings in small clusters that are scattered across the brain. True findings in imaging data, however, usually are in sufficiently large contiguous regions on the order of at least several cubic millimeters because brain processes are supported by regions that have finite spatial extent and also because of the inherent smoothing due to acquisition and processing of the imaging data.

Topological FDR procedures (Chumbley et al., 2010) were then developed in an attempt to overcome the limitations of the FDR procedure. It computes conditional *p*‐values for peaks within clusters of test statistic and thus considers the height and spatial extent for clusters of findings. It controls the number of false positive clusters rather than the number of false positive voxels. Although topological FDR substantially improves the statistical power to detect more biologically plausible clusters of effects, it requires a prespecified threshold, called a “cluster defining threshold” (CDT), to compute statistical significance of those clusters. The CDT is specified arbitrarily, and small variations in its value can have large influences on the regions declared to be statistically significant.

Threshold free cluster enhancement (TFCE) (Smith & Nichols, 2009) is intended to obviate the need to specify a CDT. It computes a transformed test statistic from the geometry of the random field assessed at several values of the CDT and then, calculates a weighted product of the cluster's spatial extent at each threshold with the test statistic value at each voxel. The significance of the transformed test statistic is evaluated using a permutation procedure at a significance level of 0.05. TFCE, however, requires specification of two weights, one for the spatial extent of a cluster and the other for the test statistic at each voxel. Probabilistic TFCE (pTFCE) (Spisak et al., 2019) and Local Indicators of Spatial Association (LISA) (Lohmann et al., 2018) were developed to compute *p*‐values at each voxel based on the local geometry of the random field without having to specify the weights for spatial extent and value of the test statistic. The probability values in pTFCE subsequently create another random field that is assessed using the theory for Gaussian random fields (GRF) (Adler, 1976, 1981; Adler & Hasofer, 1976) to calculate statistical significance of the probability values. Despite strengths of TFCE and pTFCE procedures, the transformed statistic of TFCE are difficult to interpret, the application of GRF theory to *p*‐values in pTFCE is very conservative with high false negative rates, and TFCE has low spatial specificity (Woo et al., 2014) as significant regions comprise voxels that have very small effect sizes.

To address the limitations of these prior approaches to the multiple comparisons problem of MRI datasets, we have developed a procedure that combines features of the local geometry of the random field of the test static with the *p*‐value for the test statistic, similar to FDR, but without the need to specify a CDT. The FDR procedure exploits the fact that *p*‐values have a uniform distribution under the null hypothesis. FDR however requires a sufficiently high statistical power at each voxel to detect true positives, which in turn necessitates a large number of participants for discriminating *p*‐values of the null hypothesis from those of the alternate hypothesis. Unfortunately, imaging studies often lack the voxelwise statistical power needed for effective FDR correction because of the cost, time, and effort required to recruit the requisite number of participants and to process those data. The requirement for sufficiently high effect size in the FDR procedure comes from the fact that the *p*‐values for test statistic of null hypothesis cannot be discriminated from those of alternate hypothesis. Unlike TFCE or pTFCE, we use the geometry of the random field defined by the test statistic to compute the probability that the local random field is distributed according to the null hypothesis. This geometry‐derived probability is subsequently employed to discriminate *p*‐values for the test statistics from the null and alternate hypotheses, thereby overcoming the constraints in FDR. By combining voxelwise *p*‐values with geometry‐derived probabilities, the proposed procedure provides consistently high statistical power to detect true positive findings in the data.

## 2 MATERIALS AND METHODS

The proposed geometry‐derived statistical significance (GDSS) procedure provides considerably greater statistical power for detecting true positives while limiting false positives. This procedure first computes the test statistics and their *p*‐values under the null hypothesis at all voxels across the brain. We then form a histogram to estimate empirical distribution of *p*‐values, which should have a uniform distribution if all test statistics were distributed according to the distribution under the null hypothesis. However, only the test statistics with *p*‐values close to 0.5 likely will be distributed according to the null hypothesis; on the other hand, test statistics with small p‐values < 0.05 could be distributed according to either the null or the alternate hypothesis (Efron, 2010). That is, the empirical distribution at small *p*‐values deviates away from the theoretical uniform distribution. The number of voxels in the histogram at *p*‐value = 0.5 therefore estimates the number voxels at small *p*‐values that are from the null hypothesis. We discriminate voxels with small p‐values as either from the null or the alternate hypothesis using the posterior probability under an Markov random field (MRF) model, which incorporates the local geometry of test statistics across voxels in the brain. A Gibbs distribution is incorporated into the MRF model to yield contiguous regions at which the null hypothesis is rejected by the proposed procedure.

### 2.1 Distribution of *p*‐values under a null hypothesis

We posit that *p*‐values across all voxels in the brain that deviate away from a uniform distribution likely represent test statistics that derive from the alternate hypothesis. Furthermore, *p*‐values from the null hypothesis can be differentiated from those of the alternate hypothesis using the local geometry of the random field that is defined by the test statistic across the entire brain. We therefore compute the probability that the local geometry at a voxel is from a random field generated under the null hypothesis. This probability value is computed using the spatial extent of the cluster and the *p*‐value for the test statistic at that voxel.

The key observation in FDR procedures is that for any density function, the *p*‐values *p* have a uniform distribution over the [0,1] domain. This is easily derived from the cumulative distribution $\mathrm{Pr}(p<p_{0})$ of *p*‐values as $\mathrm{Pr}\;\left(p\leq p_{0}\right)=\mathrm{Pr}\left[p\left(t\right)\leq p_{0}\left(t_{0}\right)\right]=\mathrm{Pr}\left[\int_{t}^{\infty }f\left(s\right)ds\leq \int_{t_{0}}^{\infty }f\left(s\right)ds\right]\;=\;\mathrm{Pr}\left[t\leq t_{0}\right]$, where f(s) is a non‐decreasing distribution function of the test statistic *s*. In other words, the cumulative distribution $\mathrm{Pr}\left(p\leq p_{0}\right)=\mathrm{Pr}\left[t\leq t_{0}\right]=\mathrm{CDF}\left(t_{0}\right)=\left(1-p_{0}\right)\;$ increases linearly as *p*_0_ decreases from 1 to 0 and *t*_0_ increases from −∞ to ∞. The density for the *p*‐values, or the derivative of $\mathrm{Pr}(p\leq p_{0})$ w.r.t *p*_0_ equals −1, which is a constant, and therefore, *p*‐values are uniformly distributed over the [0, 1] domain. The *p*‐values, however, deviate from the uniform distribution when test statistics are distributed according to two or more distinct density functions or by an empirical distribution that differs from the theoretical distribution. FDR assumes that the null distribution differs sufficiently from the alternate distribution—that is, that Cohen's D effect size at each voxel is sufficiently large. Under this assumption, FDR rejects all *p*‐values, starting from the smallest *p*‐value, until an expected number of *p*‐values from the null hypothesis have been rejected. Although FDR provides greater statistical power than FWER, FDR fails to reject the null hypothesis at voxels having a small‐to‐medium Cohen's D effect size, and even large Cohen's D effect sizes if the sample size is small.

**FIGURE 1:**
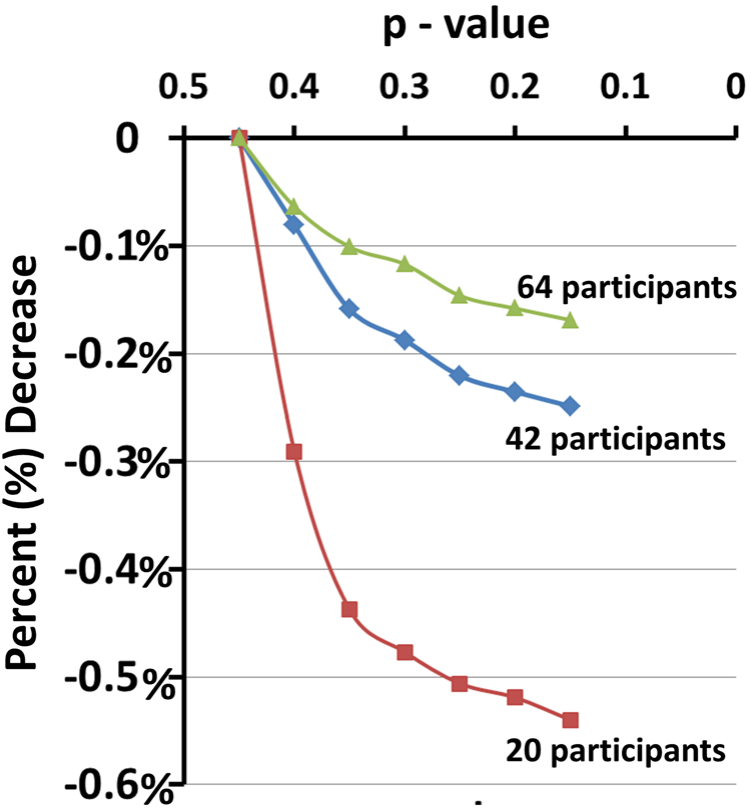
Sensitivity of the GDSS procedure to the range of p‐values for estimating the number *n*_0_ of voxels in the uniform histogram for the null hypothesis. We estimated *n*_0_ as the average number of voxels in each bin of the histogram by averaging the number of voxels from *p*‐value 0.5 to either 0.45, 0.40, 0.35, 0.30, 0.25, 0.20, or 0.15 in three subcohorts with either 20, 32, or 64 participants from our real‐world dataset. The estimated *n*_0_ was used in the GDSS procedure to reject the null hypotheses. We counted the number of voxels at which the null hypothesis was rejected and calculated the percent change in that number relative to the number for rejected null hypothesis for *p*‐value range (0.5, 0.45). The estimated *n*_0_ increases as the average is computed over smaller *p*‐values; therefore, the null hypothesis is rejected at fewer voxels by the GDSS procedure. The graphs show that for each of the three subcohorts of our real‐world dataset, the percent decrease in rejection of null hypothesis is rapid up to *p*‐value = 0.35 but then decreases more slowly. Nevertheless, the percent decrease in the number voxels at which the null hypothesis is rejected is smaller than 0.6%, thereby suggesting that the GDSS procedure has low sensitivity to the range of *p*‐values used to estimate *n*_0_

We also note that voxels with *p*‐values near 0.5 are likely from the null hypothesis, (Efron, 2010) and therefore, the number *n*_0_ of voxels in the histogram of *p*‐values at 0.5 equals the number of voxels at other *p*‐values, if the histogram has a uniform distribution of *p*‐values. Statistical models such as generalized linear regression model (GLM) are applied to data at each voxel for computing a test statistic that assesses how the brain measure is associated with an outcome measure. *p*‐values for that test statistic are computed using a theoretical distribution that has mean value equal to zero under the null hypothesis; that is, null hypothesis states that brain measure is not associated with outcome measures. The empirical null hypothesis for real‐world data may have a mean that differs from, even though it is close to, zero. The number of voxels at a *p*‐value = 0.5 therefore do not accurately estimate the number of voxels from the null hypothesis. Therefore, to account for the fact that the empirical null may differ from the theoretical null distribution, and to estimate the number of voxels under the null hypothesis, we computed the average of the number of voxels with *p*‐values in the range of 0.35 to 0.5 to estimate *n*_0_, the number of voxels where the statistic is distributed according to the null hypothesis. We estimated *n*_0_ for *p*‐values from 0.35 to 0.5 because our real‐world dataset showed rapidly increasing number of voxels for *p*‐values > 0.3 because, for decreasing *p*‐values, increasing fraction of voxels are distributed according to the alternate hypothesis. Therefore, only the central region of the null hypothesis has been previously used to estimate fraction of voxels from the null hypothesis (Efron, 2010, Chapter 4). Including smaller *p*‐values would overestimates *n*_0_, thereby leading to rejection of the null hypothesis at fewer voxels. We simulated how the use of smaller *p*‐values to estimate *n*_0_ decreases the number of voxels at which the null hypothesis is rejected in three subsets of our real‐world dataset (Figure 1). These simulations showed that, as expected, the proposed GDSS procedure rejected the null hypothesis at fewer voxels for smaller *p*‐values. However, the percentage of decrease is small (less than 0.6%) and the decrease saturates for *p*‐values smaller than 0.35. That is, the GDSS procedure has low sensitivity for the range of *p*‐values used to estimate *n*_0_. Nevertheless, even though range of *p*‐values from 0.35 to 0.5 suffices for our real‐world dataset, this range ideally can be selected from the histogram of *p*‐values in other datasets.

### 2.2 Geometry‐derived statistical significance

#### 2.2.1 Ascertaining statistical significance using GDSS

Our procedure therefore first (a) computes *p*‐values for test statistic under the hypothesized, theoretical null distribution; (b) forms a histogram for those *p*‐values at all brain voxels; (c) averages the number of voxels in *p*‐value bins from 0.35 to 0.5 to compute the average number of voxels *n*_0_ within each bin. We estimated *n*_0_ by computing the average number of voxels over *p*‐values from 0.35 to 0.5 because the empirical null distribution may not be centered at zero mean; (d) computes the geometric probability $p_{H_{0}}\left(x\right)$ that the test statistic at a voxel is from a random field under the null hypothesis by accounting for the spatial extent of a cluster comprising the voxel and using its test statistic value; (e) computes the geometric probability by varying CDT (i.e., z‐scores) from 2.0 to 6.5 or one‐sided *p*‐value of 0.02 to 5*10^−5^; and (f) combines the geometric probabilities for the null random field with *p*‐values in the histogram, such that in each bin *n*_0_ voxels with the largest geometric probability are assigned to the null hypothesis, and the null hypothesis is rejected at all other voxels in the bin. This procedure therefore ascribes statistical significance to a voxel by combining both the *p*‐value of the voxel computed under the null hypothesis and the probability value using the geometry of the random field. This GDSS procedure yields regions of significant statistical effect that comprise isolated voxels where the null hypothesis is not rejected. We regard these isolated voxels as biologically implausible and ascribe their presence to noise in the imaging data. We therefore model the spatial distribution of significant voxels as a MRF (Li, 1995), use Gibbs distribution (Besag, 1974a) to calculate a priori probability and yield an assignment that maximizes the a posterior probability (Li, 1995). GDSS therefore maximizes the probability of assigning statistical significance to voxels with true positive findings by combining their *p*‐values with the geometry‐derived probability for the surrounding cluster in a random field under the null hypothesis.

#### 2.2.2 Geometry‐derived probability under the null hypothesis

Let $p_{H_{0}}\left(x\right)$ be the probability that the voxel *v* takes a value *x* in a random field where the test statistic is distributed under the null hypothesis. Let *C* be a cluster that encompasses the voxel at a CDT of *u*. We compute the probability $p_{H_{0}}\left(x\right)$ for voxel *v* taking a value *x* when the test statistic is distributed under the null hypothesis *H*_0_ as:

$$p_{H_{0}}\;\left(x\right)=\sum_{C,u}p\left(x,C,u\right)\;=\sum_{u}\sum_{C}p\left(C|x,u\right)\cdot p\left(x|u\right)\cdot p\left(u\right)\;$$

where p(C|x,u) is the probability that a cluster *C* of size *k* encompasses the voxel *x* at a threshold *u*; p(x|u) is the probability that the random field will exceed *x*, given that it has exceeded the threshold *u*; and p(u) is the prior probability of threshold *u*. We varied the threshold *u* from 2.0 to 6.5, assuming a uniform prior probability p(u) for the threshold *u*. An a priori distribution other than a uniform distribution may reduce false positives possibly without substantially affecting true positives in the findings. For simplicity of the presentation, we do not use the subscript *H*_0_ in the following equations. We compute the probability p(C=k|x,u) for the spatial extent *k* (i.e., the total number of voxels) of the cluster *C* as (Friston et al., 1994; Worsley et al., 1996) $P\;\left(C\;=\;k|x,u\right)=\frac{2\beta }{D}\;\cdot k^{\left(\frac{2}{D}-1\right)}\cdot \exp\left(-\beta k^{2/D}\right)$, where $\beta \;={\left[\Gamma \left(\frac{D}{2}+1\right)\cdot E\left(\chi_{u}\right)/E\left(N\right)\right]}^{2/D}\;$, *N* is the number of locations in the brain with values greater than the threshold *u*, *D* is the dimensionality of the random field (which equals 3 in our experiments), and $E(\chi_{u})$ is the expected Euler characteristic (Adler, 1976, 1977, 2010; Adler & Hasofer, 1976; Hasofer, 1978) for a *D*‐dimensional random field. For a smooth random field X(t) with variance σ^2^ defined over a *D*‐dimensional volume *S*, we employed the Adler method (Adler, 1976, 1977, 2010; Adler & Hasofer, 1976; Hasofer, 1978) for computing $E(\chi_{u})$ at a threshold *u* as $E\;\left(\chi_{u}\right)=\;L\left(S\right)\cdot {\left(2\pi \right)}^{-(D+1)/2}\cdot \sigma^{-(2D-1)}{\left|A\right|}^{1/2}\cdot P\left(u\right)\cdot e^{-u^{2}/2\sigma^{2}}\;$where L(S) is the Lebesgue measure of the volume *S*, *A* is the determinant of the covariance matrix for the first‐order partial derivatives of the random field X(t) computed as difference of immediately neighboring voxel intensities, and P(u) is the Hermite polynomial (Hasofer, 1978). The conditional probability p(x|u) that the random field will exceed value *x*, given that the it has exceed value *u*, is computed as $p\;\left(x|u\right)=\frac{p(x,\;u)}{p(u)}\;=\frac{p(x)}{p(u)}\;$, which for a GRF with standard deviation σ=1 is computed as (Chumbley et al., 2010; Worsley et al., 1996): $p\;\left(x|u\right)=\left(\frac{x^{2}-1}{u^{2}-1}\right)\;\cdot \exp\left(-(x^{2}-u^{2})/2\right)$.

#### 2.2.3 A Markov random field model for significant locations

To ensure that significant findings form contiguous clusters of voxels, we assume a MRF model for locations where the null hypothesis is rejected (Besag, 1974b, 1986; Geman & Geman, 1984). An MRF model mathematically formulates the a priori belief that regions of biologically plausible effects comprise contiguous voxels, and it imposes the constraint that rejection of the null hypothesis at a voxel *v* depends on the probability of whether the null hypothesis is rejected at its neighboring voxels. We define a neighborhood as the eight immediately adjacent voxels within the same slice and one voxel in each slice neighboring the voxel *v*. In addition to whether majority of the neighboring voxels are significant, the probability that a voxel is significant is a function of its *p*‐value relative to the *p*‐values of other significant and non‐significant voxels in its *p*‐value histogram bin. For each bin, we computed the mean and variance for the *p*‐values for both significant and non‐significant voxels. Therefore, if $S\;=\;[s_{1},\;s_{2},\text{…},\;s_{N}]$ is an assignment of significance $s_{i}$ to each of the *i* voxels in the brain and $Y\;=\;[y_{1},\;y_{2},\;\text{…},y_{N}]$ are their *p*‐values, we computed the posterior probability $P_{S|Y}\left(s|y\right)$ for the geometric distribution of significant *p*‐values and assigned statistical significance to the voxels on the domain Ω in the brain such that the configuration $\hat{s}$ maximizes the posterior probability.

$$\;\hat{s}=\arg\max_{s\in \Omega }P_{S|Y}\left(s|y\right)\;=\arg\max_{s\in \Omega }P_{Y|S}\left(y|s\right)\cdot P_{S}\left(s\right)\;$$

The posterior probability $P_{S|Y}\left(s|y\right)$ is computed as the product of the likelihood probability $P_{Y|S}\left(y|s\right)$ and the a priori probability $P_{S}\left(s\right)$, which is evaluated, assuming a Gibbs distribution, for each voxel in every bin *b* of the histogram of *p*‐values. The probability $P_{Y}\left(y\right)$ of the *p*‐values across the brain is independent of voxels where the null hypothesis is rejected and therefore is not included in the optimization procedure for estimating $\hat{s}$. We computed the likelihood probability as $P_{Y|S}\;\left(y|s\right)=\exp\left(-\sum_{i=1,2}\left[\frac{{\left(y-\mu_{i}\right)}^{2}}{2\sigma_{i}^{2}}+\frac{1}{2}\cdot \ln\left(2\pi \sigma_{i}^{2}\right)\right]\right)\;$, where μ_1_ and σ_1_ are the mean and standard deviation for *n*_0_ *p*‐values in a bin *b* that have the largest metric probability $p_{H_{0}}\left(x\right)$ and, hence, are presumed to derive from the null hypothesis; whereas μ_2_ and σ_2_ are the mean and standard deviation for the rest of the *p*‐values in the bin *b* that are presumed to derive from the alternate hypothesis. The Gibbs distribution for the a priori probability $P_{S}\left(s\right)$ is computed as $P_{S}\left(s\right)\propto \;\exp\left(-\sum_{c\in C}V_{c}\left(s\right)\right)=\frac{1}{Z}\;\cdot \exp\left(-\sum_{c\in C}V_{c}\left(s\right)\right)$, where *C* is the set of all cliques *c* determined by the four immediately neighboring and four diagonal voxels within the slice and the two immediately neighboring voxels in the two adjacent slices, $V_{c}\left(s\right)$ is the potential energy for the configuration *s*, and $Z\;=\sum_{s\in \Omega }\exp\left(-\sum_{c\in C}V_{c}\left(s\right)\right)\;$ is the partition function over all possible configurations *s*.

### 2.3 Comparator procedures

#### 2.3.1 False discovery rate

FDR (Benjamini & Yekutieli, 2001) first sorts the *N* *p*‐values $P_{1},\text{…},\;P_{N}$ by their increasing order $P_{\left(1\right)},\;\text{…},\;P_{\left(N\right)}$ and then, for a specified FDR α, finds the largest integer *k* such that $P_{\left(k\right)}\leq \frac{k}{N\cdot c(N)}\alpha$, where $c\;\left(N\right)=\sum_{i=1}^{N}\frac{1}{i}\;$. All *p*‐values smaller than $P_{\left(k\right)}$ are deemed to be statistically significant and to warrant rejection of their null hypotheses.

#### 2.3.2 Topological FDR

Topological FDR (Benjamini & Yekutieli, 2001; Chumbley et al., 2010) models the distribution of the test statistic as a random field and defines clusters of findings by thresholding the statistical map at a prespecified CDT *u*, which we set equal to 3.0. The procedure then computes the conditional *p*‐value p(x|u) for the peak statistic *x* within a cluster, assuming that the random field exceeds the threshold *u*—that is, it computes the conditional *p*‐value $p\;\left(x|u\right)=\left(\frac{x^{2}-1}{u^{2}-1}\right)\;\cdot \exp\left(-(x^{2}-u^{2})/2\right)$. Subsequently, FDR is applied to the conditional *p*‐values at a FDR = 0.05 to compute a *p*‐value $P_{\left(k\right)}$ such that all conditional *p*‐values smaller than $P_{\left(k\right)}$, and all voxels within their associated clusters, are considered to be statistically significant.

#### 2.3.3 Threshold free cluster enhancement

The spatial extent of the statistically significant regions determined by topological FDR requires specifying a CDT. TFCE (Smith & Nichols, 2009) overcomes this constraint by first transforming the test statistics using the local geometry—the spatial extent—of clusters at varying CDT values of *u* and the test statistic in the random field, and then applying a nonparameteric, permutation‐based procedure to assess the significance of the transformed test statistics. We computed the transformed statistical map and applied the nonparametric permutation within SPM12 (Ashburner, 2012).

#### 2.3.4 Probabilistic TFCE

pTFCE applies Bayes’ rule to compute a conditional probability from the test statistic at a voxel and cluster size that contains that voxel. The conditional probabilities are aggregated for varying cluster‐forming thresholds, thereby defining a map of probabilities and corresponding *z*‐statistic field across the brain. We used SPM12 (Ashburner, 2012) to implement pTFCE and to compute probability maps for all our datasets. We subsequently applied to all synthetic data the GRF theory for determining statistically significant regions (Spisak et al., 2019). In the real‐world data analyses, pTFCE‐generated probability values did not survive GRF correction, except for one analysis in which the application of GRF was equivalent to thresholding the probability map at –logP = 13.5, which is almost the same as –logP = 13.6 previously employed, (Spisak et al., 2019) We therefore present all pTFCE findings by thresholding the pTFCE‐generated probability maps at −logP = 13.5.

### 2.4 Experiments

We evaluated whether GDSS correctly rejects the null hypothesis by computing (a) the true positive rate (TPR; i.e., statistical power) and (b) the false positive rate (FPR, i.e., type‐I error) in both synthetic data generated with known amounts of added signal and real‐world data that had robust associations of regional cerebral blood flow (rCBF) with the age of the participants. We compared the performance of the proposed method with the performance of each of the comparator procedures (FDR, topological FDR, TFCE, and pTCFE).

#### 2.4.1 Synthetic datasets

We generated synthetic data in a three‐dimensional space of 100×100×100voxels in 50 participants. Because MRI studies typically evaluate the associations of an independent variable with brain measures, we assumed that variable was age (*x*) of the participants, with a mean age ($\mu_{x}$) of 20 years with a standard deviation ($\sigma_{x}$) of 2 years: that is, voxel signal $y_{i}$ for the *i*th participant was modeled as *y_i_* = *β*_0_ + *β*_1_ * *x_i_* + *∈* *_i_*. The strength of association β_1_ of age with the voxelwise signal was increased in steps of 0.005 from 0.005 to 0.055. We generated a total of four synthetic datasets each for 50 participants of age selected at random from the Gaussian distribution: (1) the first dataset was generated by adding signal in a spherical region of radius 5 voxels. This dataset was generated to evaluate TPR and FPR of the various procedures for correctly rejecting the null hypotheses in small regions. (2) The second dataset was generated by adding signal in two spherical regions each of radius 5 voxels such that the two spherical regions were just touching each other. This dataset was generated to evaluate how smoothing of the imaging data affects TPR and FPR when the regions with added signal are much closer proximity than FWHM of the smoothing kernel. (3) The third dataset was generated by adding signal in three spherical regions each of radius 5 voxels such that the two regions were just touching the region in the middle. Smoothing of this imaging data will symmetrically smooth the signal in the center, thereby significantly increasing its signal to noise ratio relative that for the two neighboring spherical regions. Such regional configurations occur in real‐world dataset where true findings in neighboring regions may disproportionately increase the statistical significance of a neighboring region. We expected the TPR to increase for increasing numbers of regions with signal. We also expected that FPR would increase for increasing amount of smoothness as the signal would spread to voxels in the neighborhood of the regions with added signal. (4) The fourth dataset was generated by adding signal in a spherical region of radius 10 voxels. A region with radius of 10 voxels is eight times larger than a region with radius of 5 voxels, and therefore, permits us to ascertain the TPR and FPR in the presence of large amounts of signal in the data. Furthermore, smoothing would spread the signal across greater number of voxels neighboring the spherical region, thereby increasing FPRs and providing guidance for selecting the amount of smoothness that balances TPR and FPR across the various statistical procedures. In each of these synthetic datasets the regions with known amounts of signal were added at the center of the imaging volume to minimize the effects of smoothing across the boundaries.

We added Gaussian white noise $\varepsilon_{i}$ having a mean ($\mu_{\varepsilon })\;=\;0$ and standard deviation $(\sigma_{\varepsilon })=\;1$ to all voxels in every imaging volume. Signal within the spherical region therefore can be described using a regression model $y_{i}=\;\beta_{0}+\beta_{1}*x_{i}+\varepsilon_{i}$, where $\varepsilon ∼N(0,\sigma_{\varepsilon })$ is Gaussian‐distributed white noise, i=1,…,50 are the participants, $x_{i}∼N\left(20,\;2\right)$ is the age of the participants, and $y_{i}$ is the brain measure at a voxel in the imaging volume. The simulated data were subsequently smoothed using a Gaussian kernel with full width at half maximum (FWHM) of either 2, 4, 7, or 10 voxels. In these smoothed simulated data, we then tested the null hypothesis $H_{0}:\;\beta_{1}=\;0$ and computed a one‐sided *p*‐value under the alternate hypothesis $H_{1}:\beta_{1}>0$. We computed one‐sided *p*‐values because our alternate hypothesis was one sided. However, the proposed GDSS procedure can be applied to a histogram for two‐sided *p*‐values. If *b*_1_is the estimated value for β_1_and $s^{2}\;\left(b_{1}\right)=\frac{\sigma_{\varepsilon }^{2}}{\sum_{n}{\left(x_{i}-\bar{x}\right)}^{2}}\;=\frac{\sigma_{\varepsilon }^{2}}{\left(n-1\right)\cdot \sigma_{x}^{2}}\;$ is its squared standard error, then the corresponding test statistic $t\;=\frac{b_{1}}{s(b_{1})}\;$. The null hypothesis is rejected at the significance level of α if t>t(1−α;n−2), where t(1−α;n−2) is computed for the central Student *t*‐distribution. If $\beta_{1}=\beta_{1}^{0}\;$ is the true value for the coefficient β_1_, then the statistical power (Neter et al., 1983) $\underset{\delta }{\mathrm{Pr}}\left\{t>t(1-\alpha ;n-2)\right\}$ for rejecting the null hypothesis is computed using the noncentral Student *t*‐distribution with noncentrality measure $\delta \;=\frac{|\beta_{1}^{0}|}{s(b_{1})}\;$. The statistic t(1−α;n−2) is computed for the null hypothesis using the central *t*‐distribution such that all *t* values greater than t(1−α;n−2) are rejected at a significance level α=0.05 for n=50. Therefore, in the central *t*‐distribution, the region for rejecting the null hypothesis is all *t* values greater than t(0.95,48)=1.68. For the simulation parameters $\sigma_{x}^{2}=\;4$, $\sigma_{\varepsilon }^{2}=\;1$, and n=50, the squared standard error $s^{2}\;\left(b_{1}\right)=\frac{\sigma_{\varepsilon }^{2}}{\sum_{n}{\left(x_{i}-\bar{x}\right)}^{2}}\;=\frac{\sigma_{\varepsilon }^{2}}{\left(n-1\right)\cdot \sigma_{x}^{2}}\;=\frac{1}{(50-1)*4}\;=\;0.005$ and the noncentrality parameter $\delta \;=\frac{|\beta_{1}^{0}|}{s(b_{1})}\;$, which increases for increasing values of $\beta_{1}=\beta_{1}^{0}\;$ at t(0.95,48)=1.68. Therefore, as β_1_ increases from 0.005 to 0.055, the corresponding statistical power to reject the null hypothesis at each voxel increases from 0.057 to 0.171 in the unsmoothed data (Table 1). We chose the maximum β_1_ value of 0.055 because Gaussian smoothing with a FWHM = 4 voxels reduced the standard deviation for noise to 0.070711. The reduced Gaussian noise provided sufficient statistical power to detect the added signal within the center of the spherical region, even though statistical power was 0.171 in the unsmoothed data for this β_1_ value (Table 1). This range of statistical power therefore permits comparison of statistical procedures when applied to real‐world data where spurious associations between imaging data and putative design matrices could be present by chance (Eklund et al., 2016). We note, however, that smoothing lowers the statistical power at the boundaries of the smoothed spherical regions because of averaging across voxels with and without the added signal.

**TABLE 1:** Statistical power of association of “age” with image intensity in simulated data

Coefficient ($\beta_{1}^{0}$)	Noncentrality (δ) parameter (s(b1) = 0.0707)	Power (percentile = 1.68; DOF = 48)	Power at the center of the smoothed data
0.005	0.0707	0.057	0.254
0.0075	0.106066	0.0614	0.567
0.010	0.1414	0.065	0.627
0.0125	0.17677	0.0702	0.793
0.015	0.2121	0.075	0.905
0.0175	0.247487	0.0802	0.964
0.020	0.282843	0.085	0.989
0.050	0.707107	0.171	1.0

First column: The coefficient of association for “age” with image intensity when generating the simulated data. Second column: The noncentrality parameter for the Student's *t‐*distribution under the alternate hypothesis. Third column: The statistical power available to reject the null hypothesis in the unsmoothed data at a significance level of 0.05. Fourth column: The statistical power available to reject the null hypothesis at the center of the spherical region in simulated data. The data were smoothed using a Gaussian kernel with FWHM = 4 voxels.

#### 2.4.2 Synthetic data without signal

To compare performance of these procedures in the absence of signal, we generated simulated data whose voxel intensities were randomly distributed and were not associated with age. These data would allow us to study whether any of the procedures produce false positives and, if so, estimate spatial extent of those false positive regions. The spatial extent of false positive regions are difficult to infer from simulated data with added signal because in those data false positives largely are due to smoothing of signal to voxels that the spatially contiguous to the regions with added signal. Furthermore, we use these data to compute FWER (i.e., the probability of rejecting one or more null hypotheses when in fact the null hypothesis is true across the entire family of tests) even though the proposed GDSS and the comparator procedures do not control for FWER. The computed FWER would allow us to understand whether false positive findings are always generated by these procedures.

We generated 250 datasets each with 50 participants without adding any signal in the imaging volumes. The intensity at every voxel in each of the 250 × 50 = 12,500 imaging volumes was Gaussian white noise *∈* *_i_* with a mean (*μ_∈_*) = 0 and standard deviation (*σ_∈_*) = 1. These 12,500 volumes were subsequently smoothed by applying a Gaussian filter with FWHM = 4, 7, or 10 voxels, thereby generating a total of 50,000 volumes with varying amounts of smoothing. We then applied linear regression model to test whether image intensity was associated with age in each set of 50 participants. We expected that null hypotheses will not be rejected at any voxel in the imaging volume because voxel intensities were not associated with age. We then estimated FWER as the fraction of datasets out of 250 datasets in which the null hypothesis was rejected at one or more voxels. Imaging data were defined on a 100 × 100 × 100 voxels in 3D space and, therefore, there was significantly high probability that the null hypothesis would be rejected by chance at one or more voxels. We expected that the FWER would be high for the multiple comparisons procedures, which would imply poor control for false positives. However, even with high FWER, procedures for multiple comparisons can be effective in controlling false positives if the null hypothesis is rejected incorrectly at only very few voxels. We therefore also computed the average number of voxels at which the null hypothesis was rejected by averaging across the 250 simulated datasets that had one or more false positives. We expected that even though FWER would be high, the null hypotheses will be rejected at only small regions of contiguous voxels, and that these regions will be distributed sparsely across the entire imaging volume.

#### 2.4.3 Real‐world dataset

The ground truth for the statistical significance of associations of brain imaging measures with an independent variable is unknown in real‐world data. Therefore, performance of a statistical procedure should be evaluated using MRI data that is known to have robust associations with the independent variable to support the evaluation of performance and comparison with the performance of comparator procedures. We therefore selected real‐world perfusion MRI data for which prior analyses showed robust associations rCBF with age (Peterson et al., 2019), which survived the correction using the conservative FDR procedure at the FDR of 0.05.

##### 2.4.3.1 Participants

We acquired blood perfusion data in 43 participants with Autism Spectrum Disorder (ASD) (age = 23.31 ± 15.57 years, 31 males) and 65 typically developing (TD) participants (age = 21.64 ± 10.85 years, 49 males). The ASD participants did not differ from the TD participants on age (two‐tailed *p* = 0.19, *t* = 1.32, df = 106, using a two‐sample *t*‐test) or sex (two‐tailed *p* = 0.768, chi squared = 0.087, df = 1). The detailed demographic and clinical measures for the ASD and TD participants are presented elsewhere (Peterson et al., 2019).

##### 2.4.3.2 Arterial spin labeling data

The perfusion data were acquired on a 3T GE scanner using pulsed arterial spin labeling (PASL) that employed a PICORE (Proximal Inversion with Control for Off‐Resonance Effects) QUIPSS II (Quantitative Imaging of Perfusion using a Single Subtraction) pulse sequence (Wong et al., 1998). A 9 cm tagging slab was placed 16 mm below the proximal edge of the imaging volume. Control images were acquired using an off‐resonance adiabatic hyperbolic secant RF pulse with the same frequency offset used for the labeled images. Use of this off‐resonance RF pulse controlled for off‐resonance effects of the inverse pulse used for the labeled images. Images were acquired using a single‐shot, gradient‐echo, echo planar imaging (EPI) sequence with time to QUIPSS saturation $TI_{1}\;$= 600 ms and an inversion time of the first slice $TI_{2}$ = 1300 ms. Other sequence parameters were TE (echo time)/TR (repetition time) = 24/2300 ms, flip angle = 90°, slice thickness = 6 mm, 18 slices acquired sequentially in the inferior to superior direction, inter‐slice spacing 0.5 mm, FOV = 24 cm, and a 64 × 64 matrix, providing a nominal spatial resolution of 3.75 × 3.75 × 6.5 mm. Each scan acquired 151 imaging volumes with 5 dummy scans in 5 min 59 s. For quantification, we also acquired an M_0_ scan using gradient‐echo EPI and a TR = 15 s, at the same resolution and slice position as the arterial spin labeling (ASL) scan. White matter magnetization *M*_0wm_ was measured for absolute quantification of rCBF (Wong et al., 1998). A T1‐weighted image with high in‐plane resolution positioned in the same slice locations as the ASL data was also acquired for coregistering ASL data across participants. The localizer image was acquired using a 2D, fast spin echo sequence having TR = 2150 ms, TE = 9.94 ms, in‐plane resolution = 0.94 × 0.94 mm^2^, slice thickness = 6.5 mm, number of slices = 18, echo train length = 9, and flip angle = 90^o^.

##### 2.4.3.3 Processing the ASL data

We first corrected head motion by coregistering the PASL and *M*_0_ images to the first PASL image for each participant. The coregistered PASL images were spatially smoothed using a Gaussian kernel with FWHM = 6 mm. We then created a brain mask by averaging the PASL images for each participant. A voxelwise map for rCBF was computed from the PASL and *M*_0_WM_ data using in‐house software: control images were pairwise subtracted from the labeled images and then the subtracted images were averaged. rCBF at each voxel was calculated from the average data as $\mathrm{rCBF}\;=\;\frac{6000*\Delta I}{2\alpha *M_{0\_B}*TI_{1}*exp\left(-TI_{2}/T_{1\_B}\right)}$, where ΔI is the averaged image difference; ${\mathrm{TI}}_{1}=\;600\;\mathrm{ms}$ is the time to QUIPSS saturation; ${\mathrm{TI}}_{2}=\;1300\;\mathrm{ms}$ is the inversion time of the first slice, which is slice time corrected for other slices in the imaging volume; and *T*_1B_ = 1664 ms is the *T*_1_ of blood (Lu et al., 2004); *α* = 0.9 is the tagging efficiency; *M*_0_B_ is the MR signal from a voxel filled with arterial blood, estimated from the *M*_0_WM_ map as $M_{0\_B}=rM_{0\_WM}e^{(1/T_{2WM}-1/T_{2B})TE}$ where *r* = 1.06 is the proton density ratio of blood; and *T*_2WM_ = 70 ms, and *T*_2B_ = 200 ms (Alsop & Detre, 1996; Jarnum et al., 2010; Wong et al., 1998). rCBF maps were calculated in the imaging space of the localizer images. These maps were then normalized into the coordinate space of a single TD participant (template image) by first rigidly transforming the localizer image of each participant to their corresponding T1w anatomical image, such that the mutual information (Viola, 1995) between the two images was maximized. Each participant's T1w image was then normalized to the T1w template by applying first a similarity (Viola, 1995) and then a nonlinear transformation based on fluid flow (Christensen et al., 1997). The template image was from a TD participant whose brain was determined by a series of statistical procedures to be most representative of the brains of all TD participants (Peterson et al., 2009).

##### 2.4.3.4 Subsamples of the real‐world dataset

The statistical power to detect significant associations for a fixed effect size is proportional to the number of participants in the sample. Therefore, to evaluate the performance of the proposed method at varying levels of statistical power, we established five subsamples by randomly selecting participants from the entire cohort of 108 participants: (1) 10 ASD and 10 TD, (2) 21 ASD and 21 TD, (3) 32 ASD and 32 TD, (4) 43 ASD and 43 TD, and (5) the entire cohort of 43 ASD and 65 TD participants. Because FDR is the most conservative of the comparator procedures we evaluated, and because 108 participants provide reasonable statistical power, findings that survived FDR correction in this cohort of 108 participants were regarded as true positives for the associations of rCBF with age. We however note that 5% of these findings nevertheless are expected to be false positives. We used these findings to compare the performance of all statistical procedures applied separately to the five participant subsamples. We assumed that the effect size for the association of rCBF with age does not vary with the number of participants, though effect size could in reality vary by chance and based on variability in the clinical and demographic characteristics that could influence rCBF in the five subsamples. We therefore assessed how effect size and statistical power varied with sample size.

## 3 RESULTS

### 3.1 Maximum a posterior assignment using an MRF model

**FIGURE 2:**
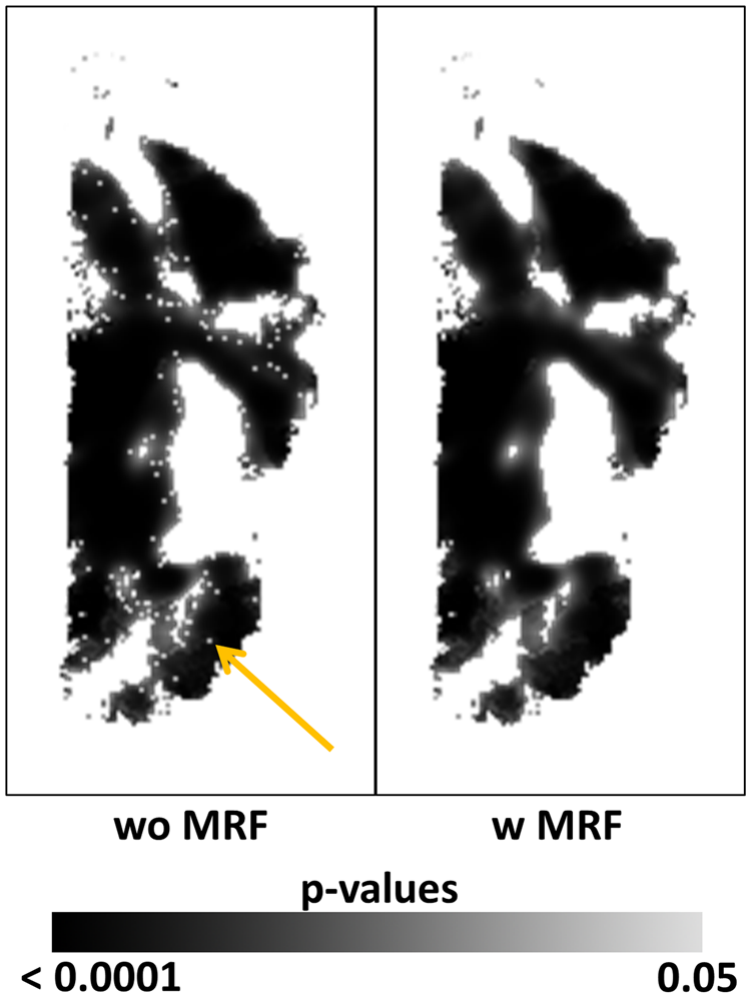
Maximum a posterior (MAP), Markov random field model for rejecting the null hypothesis. We used geometry‐derived statistical significance (GDSS) to identify significant associations of regional cerebral blood flow (rCBF) with age in our real‐world cohort of 108 participants (65 typically developing and 45 participants with Autism Spectrum Disorder). A multiple linear regression model included rCBF as the dependent variable, age as the independent variable, and sex, diagnosis, and full scale IQ as covariates. The null hypothesis was that the age was not associated with rCBF, whereas the alternate hypothesis was that rCBF decreases with age. Our previous analyses showed that the age effects on rCBF did not differ by diagnosis. GDSS was applied both without (Left) and with (Right) the use of an MRF model to account for the geometry (spatial extent) of regions with significant associations. Left: Without the use of MRF, the large region with statistically significant associations had numerous isolated voxels (an example voxel is indicated by the arrow) where the null hypothesis was not rejected. Right: The use of MRF with a Gibbs distribution as an a priori probability yielded a contiguous region of voxels that maximized the a posterior probability of rejecting the null hypothesis. The colorbar shows the grayscale encoding of the *p*‐values. MRF, Markov random field; w, with; wo, without

Without application of the MRF model, large regions with statistically significant associations contained numerous isolated voxels where the null hypothesis was not rejected (Figure 2, left). Application of the MRF model, however, eliminated those isolated voxels to yield spatially contiguous regions of statistical significance (Figure 1, right). That is, the MRF model generated significant regions that are biologically plausible. We therefore present all findings for the proposed methods with the use of MRF model.

### 3.2 Synthetic datasets

#### 3.2.1 Spherical regions of radius 5 mm

**FIGURE 3:**
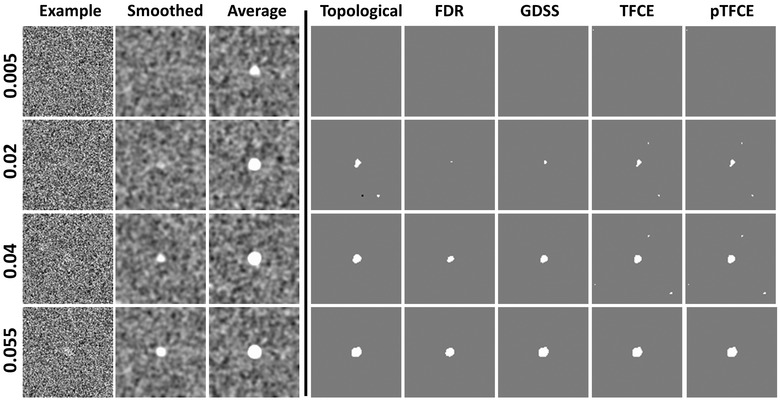
Synthetic data with known signal within a spherical region of radius five voxels. We generated simulated data for 50 participants in which within a spherical region the associations of image intensity with age increased from 0.005 to 0.055 in increments of 0.005. These data were simulated over a 3D domain of 100 × 100 × 100 voxels. This yielded 11 datasets, each with 50 participants. We subsequently added Gaussian white noise with mean of 0 and standard deviation of 1, yielding a statistical power for the added signal increase from 0.057 to 0.189. The age for each participant was randomly selected from a Gaussian distribution with mean ($\mu_{x}$) of 20 years and standard deviation ($\sigma_{x}$) of 2 years. The simulated data were smoothed using a Gaussian kernel with FWHM = 4 voxels. We then applied the various statistical procedures for detecting statistically significant associations using a linear regression model: $y_{i}=\beta_{0}\;+\beta_{1}*age_{i}+\varepsilon_{i}$, for i=1,…,50. Column “Example”: A slice through the simulated data before Gaussian smoothing. Column “Smoothed”: The same slice after Gaussian smoothing. Smoothing, and hence averaging of noise, significantly increased the statistical power compared with unsmoothed data to detect true positives at the center of the sphere. Column “Average”: The same data after averaging across the 50 participants. Columns “Topological,” “FDR,” “GDSS,” “TFCE,” and “pTFCE”: The map of voxels at which the null hypothesis was rejected by each of the five methods to detect statistically significant associations while controlling for multiple comparisons. Topological FDR was applied at a cluster defining threshold of 3.0, and the TFCE and pTFCE maps were computed in SPM12. Each row presents findings for increasing strength of association of image intensity with age. At $\beta_{1}=\;0.005$, (first row), the null hypothesis was not rejected at any voxel using any of the procedures (none of them detected true positives), whereas at $\beta_{1}=\;0.055$, (last row), associations were statistically significant at most voxels within the spherical region using each of the five procedures

**FIGURE 4:**
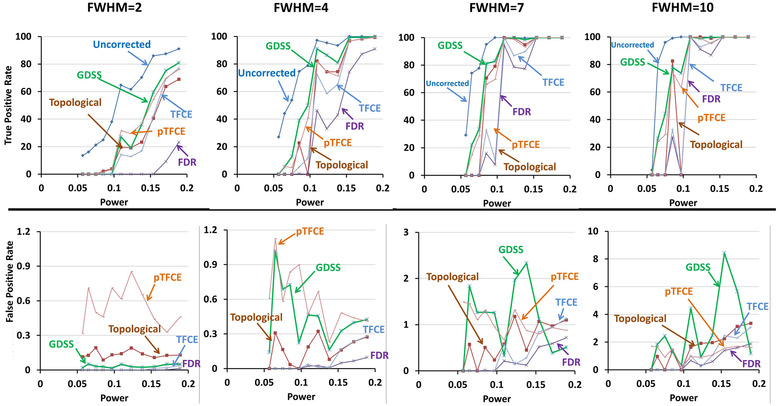
True positive rate (TPR) and false positive rate (FPR) of the statistical procedures applied to simulated data with a spherical region of radius 5 voxels. The simulated data were smoothed by applying a Gaussian filter with full width at half maximum (FWHM) of either 2, 4, 7, or 10 voxels. The *x*‐axis plots the voxelwise statistical power prior to smoothing of the simulated data. Because simulated signal comprised very few voxels, for comparisons we normalized false positive rate for a statistical procedure that controls for multiple comparisons to the false positive rate in findings uncorrected for multiple comparisons. Dark blue curve plots the TPR for procedure that compute voxelwise statistical significance at a one‐sided significance level of α=0.05 without controlling for multiple comparisons. The TPRs (top row) and relative FPRs (bottom row) are shown for GDSS (green), FDR (violet), topological FDR (dark brown), TFCE (violet), and pTFCE (light brown). Top row: TPRs increase for increasing amounts of smoothness as decreasing noise increases statistical power to detect the added signal. GDSS in general had the highest TPR among the five multiple comparisons procedures that control for false positives in multiple hypotheses testing, especially when statistical power for the added signal was low. FDR was most conservative and hence had the lowest TPR; consequently, FDR had the lowest false negative rates as well. When the added signal had sufficiently high statistical power, all procedures correctly rejected the null hypothesis (i.e., detected true positives) at all voxels with the added signal in the imaging volumes. Bottom row: FPRs increased for increasing amounts of smoothness likely because smoothing spread of the signal to voxels neighboring the region with the added signal. FDR had the lowest relative false positive rate, whereas pTFCE had the highest FPRs for smoothness at FWHM = 2 and 4 voxels. At FWHM = 7 and 10 voxels, GDSS had the highest FPRs because smoothing spread the signal, leading to the rejection of the null hypothesis at the neighboring voxels. FDR, false discovery rate; FPR, false positive rate; FWER, familywise error rate; FWHM, full width at half maximum; GDSS, geometry‐derived statistical significance; pTFCE, probabilistic TFCE; TFCE, threshold free cluster enhancement; TPR, true positive rate

**FIGURE 5:**
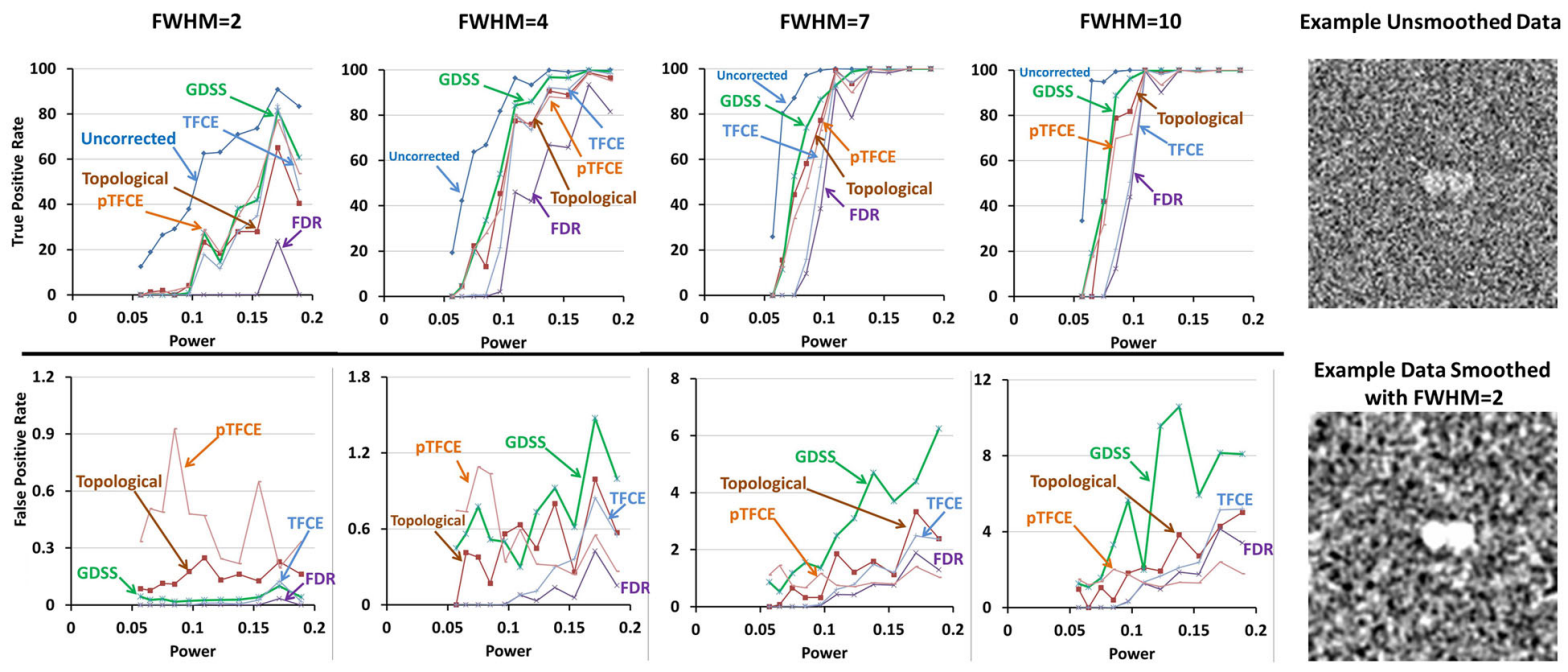
True positive rate (TPR) and false positive rate (FPR) of the statistical procedures applied to simulated data with two spherical regions each of radius 5 voxels. We smoothed the simulated data by applying a Gaussian filter with full width at half maximum (FWHM) of either 2, 4, 7, or 10 voxels. The left column shows example slices through the unsmoothed simulated data and the simulated data smoothed by applying a Gaussian filter with FWHM = 2 voxels. The *x*‐axis of the graphs plots the voxelwise statistical power prior to smoothing of the simulated data. Because simulated signal comprised very few voxels, for comparisons we normalized false positive rate for a statistical procedure that controls for multiple comparisons to the false positive rate in findings uncorrected for multiple comparisons. Dark blue curve plots the TPR for procedure that compute voxelwise statistical significance at a one‐sided significance level of α=0.05 without controlling for multiple comparisons. The TPRs (top row) and relative FPRs (bottom row) are shown for GDSS (green), FDR (violet), topological FDR (dark brown), TFCE (violet), and pTFCE (light brown). Top row: TPRs increase for increasing amounts of smoothness because noise decreases, thereby increasing statistical power to detect the added signal. GDSS in general had the highest TPR among the five multiple comparisons procedures that control for false positives in multiple hypotheses testing. FDR was most conservative and hence had the lowest TPR. When the added signal had sufficiently high statistical power, all procedures correctly rejected the null hypothesis (i.e., detected true positives) at all voxels with the added signal in the imaging volumes. Bottom row: FPRs increased for increasing amounts of smoothness likely because smoothing spread of the signal to voxels neighboring the region with the added signal. FDR had the lowest relative false positive rate, whereas pTFCE had the highest FPRs for smoothness at FWHM = 2. At FWHM = 4, 7, and 10 voxels, GDSS had the highest FPRs because smoothing spread the signal, leading to the rejection of the null hypothesis at the neighboring voxels. FDR, false discovery rate; FPR, false positive rate; FWER, familywise error rate; FWHM, full width at half maximum; GDSS, geometry‐derived statistical significance; pTFCE, probabilistic TFCE; TFCE, threshold free cluster enhancement; TPR, true positive rate

**FIGURE 6:**
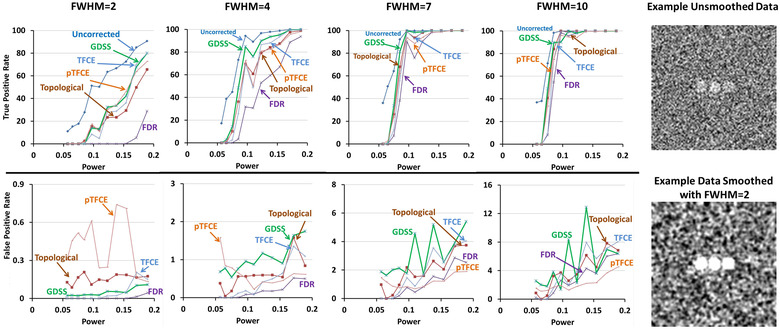
True positive rate (TPR) and false positive rate (FPR) of the statistical procedures applied to simulated data with three spherical regions each of radius 5 voxels. We smoothed the simulated data by applying a Gaussian filter with full width at half maximum (FWHM) of either 2, 4, 7, or 10 voxels. The left column shows example slices through the unsmoothed simulated data and the simulated data smoothed by applying a Gaussian filter with FWHM = 2 voxels. The *x*‐axis of the graphs plots the voxelwise statistical power prior to smoothing of the simulated data. Because simulated signal comprised very few voxels, for comparisons we normalized false positive rate for a statistical procedure that controls for multiple comparisons to the false positive rate in findings uncorrected for multiple comparisons. Dark blue curve plots the TPR for procedure that compute voxelwise statistical significance at a one‐sided significance level of α=0.05 without controlling for multiple comparisons. The TPRs (top row) and relative FPRs (bottom row) are shown for GDSS (green), FDR (violet), topological FDR (dark brown), TFCE (violet), and pTFCE (light brown). Top row: TPRs increase for increasing amounts of smoothness because noise decreases, thereby increasing statistical power to detect the added signal. GDSS in general had the highest TPR among the five multiple comparisons procedures that control for false positives in multiple hypotheses testing. FDR was most conservative and hence had the lowest TPR. When the added signal had sufficiently high statistical power, all procedures correctly rejected the null hypothesis (i.e., detected true positives) at all voxels with the added signal in the imaging volumes. Bottom row: FPRs increased for increasing amounts of smoothness likely because smoothing spread of the signal to voxels neighboring the region with the added signal. FDR had the lowest relative false positive rate, whereas pTFCE had the highest FPRs for smoothness at FWHM = 2 but the lowest FPR at FWHM = 7 and 10 voxels. On the other hand, GDSS had the highest FPRs at FWHM = 4, 7, and 10 voxels because smoothing spread the signal, leading to the rejection of the null hypothesis at the neighboring voxels. FDR, false discovery rate; FPR, false positive rate; FWER, familywise error rate; FWHM, full width at half maximum; GDSS, geometry‐derived statistical significance; pTFCE, probabilistic TFCE; TFCE, threshold free cluster enhancement; TPR, true positive rate

For the small magnitude association $\beta_{1}=\;0.005$ for image intensity with age (i.e., providing unsmoothed statistical power = 0.057 and smoothed statistical power = 0.254; Table 1), none of the statistical procedures rejected the null hypothesis at any voxel within the sphere. That is, all procedures failed to reject the null hypotheses, and therefore, their TPR were 0 at all FWHM, irrespective of whether the signal was added to one, two, or three regions ( Figures 3, 4, 5, 6). For the large magnitude association $\beta_{1}=\;0.050$ (i.e., providing unsmoothed statistical power = 0.171 and smoothed statistical power = 1.0; Table 1), all procedures except for FDR rejected the null hypothesis at every voxel within the spheres (Figures 4, 5, 6). FDR however correctly rejected all null hypotheses for FWHM = 7 and 10 when greater smoothing provided higher statistical power to reject the null hypotheses (Figures 4, 5, 6). At FWHM = 4 voxels, GDSS compared to other procedures yielded the highest TPR when statistical power was low: TPRs were 5.44, 12.81, and 39.03 for GDSS; 0, 0, and 5.24 for TFCE; and 5.82, 4.85, and 20.0 for pTFCE. FPR was highest for pTFCE at FWHM = 2 voxels, whereas was highest for GDSS at FWHM = 4, 7, and 10 voxels (Figures 4, 5, 6). These patterns of differences in TPR and FPR values across the various statistical procedures did not change when the signal was added to one, two, or three spherical regions. FPR for all procedures increased for increasing amounts of smoothness because the added signal was spread to the voxels neighboring the spherical regions, thereby leading to the rejection of the null hypotheses at those voxels. TPR was high and FPR was low at FWHM = 4 voxels, thereby suggesting smoothing with Gaussian kernel of FWHM = 4 voxels provided the optimal smoothing for correctly rejecting the null hypotheses.

#### 3.2.2 Spherical region of radius 10 mm

**FIGURE 7:**
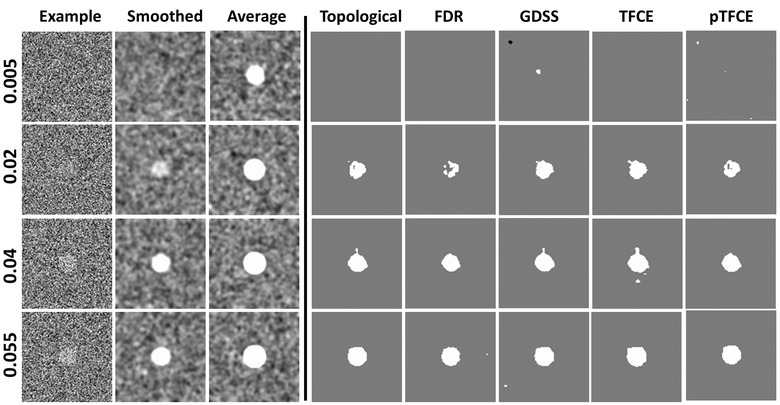
Synthetic data with known signal in a spherical region of radius 10 voxels similar to the data in Figure 2 above, synthetic data were generated for 50 participants using a known signal within a spherical region, but here with radius 10 voxels, centered in a 3D volume of 100 × 100 × 100 voxels. Image intensity within the sphere was associated with the age of the participants according to the linear relation $y_{i}=\beta_{0}\;+\beta_{1}*age_{i}$, for i=1,…,50 participants. The association β_1_ was increased from 0.005 to 0.055 in increments of 0.005. Column “Example”: A slice through the simulated data before a smoothing Gaussian kernel. Column “Smoothed”: The same slice though the simulated data after smoothing the data by a Gaussian kernel with FWHM = 4 voxels. Column “Average”: The same slice averaged across the 50 participants. Columns “Topological , ” “FDR , ” “GDSS , ” “TFCE , ” and “pTFCE”: The map of voxels at which the null hypothesis was rejected by each of the five methods to detect statistically significant associations while controlling for multiple comparisons. topological FDR was applied at a cluster defining threshold of 3.0, and the TFCE and pTFCE maps were computed using SPM12. Each row presents findings for increasing strength of association of image intensity with age. At $\beta_{1}=\;0.005$, (first row), no voxel was deemed to have statistically significant association for any procedure except GDSS, whereas at $\beta_{1}=\;0.055\;($last row), almost all voxels in the sphere were deemed to have statistically significant associations in all statistical procedures evaluated

**FIGURE 8:**
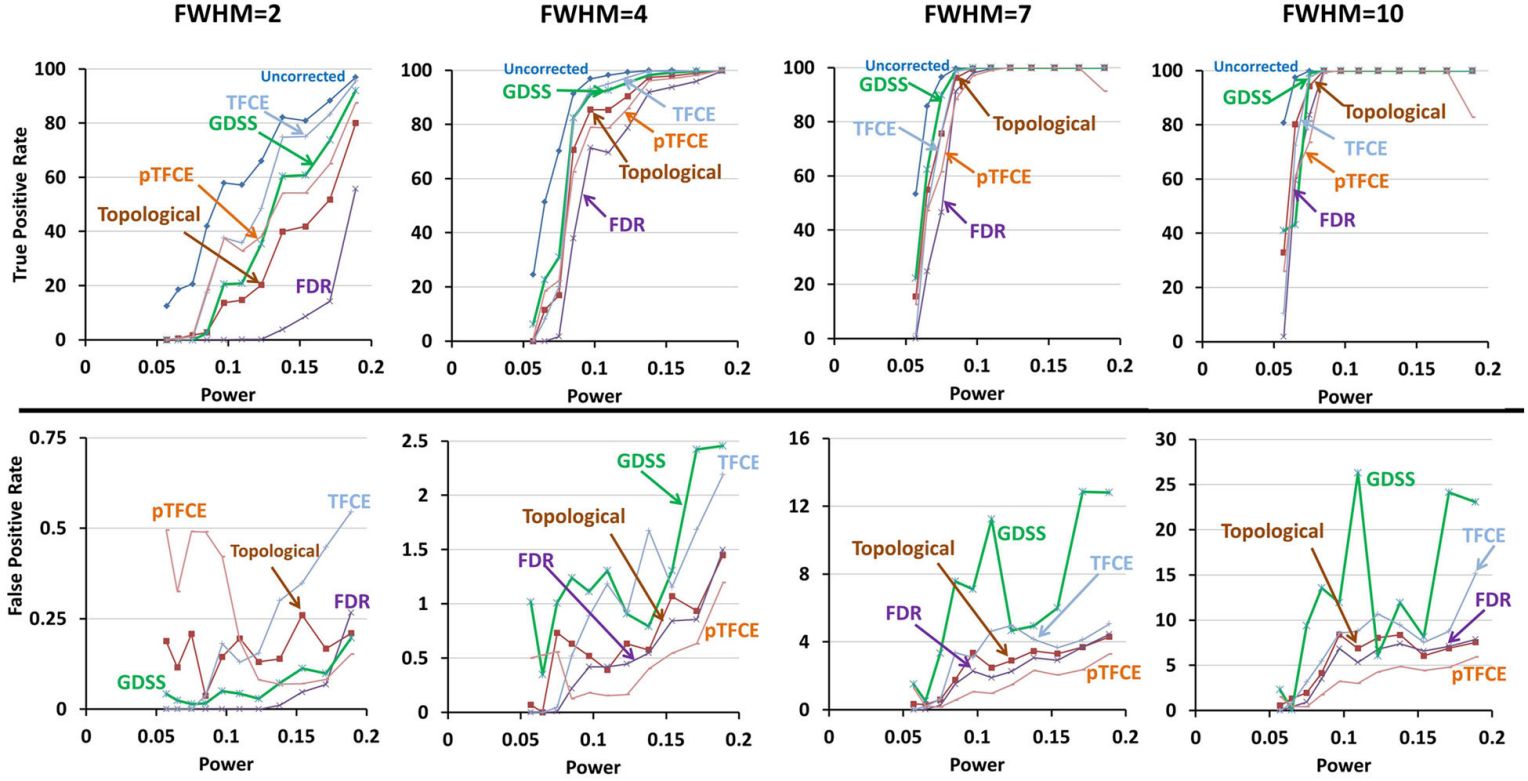
True positive rate (TPR) and false positive rate (FPR) for statistical procedures in a larger cluster The *x*‐axis plots the voxelwise statistical power for known signal added to a spherical region with radius 10 voxels at the center of the imaging volume. The simulated data were subsequently smoothed by applying a Gaussian filter with full width at half maximum (FWHM) of either 2, 4, 7, or 10 voxels. The *x*‐axis plots the voxelwise statistical power prior to smoothing of the simulated data. Because simulated signal comprised very few voxels, for comparisons we normalized false positive rate for a statistical procedure that controls for multiple comparisons to the false positive rate in findings uncorrected for multiple comparisons. Dark blue curve plots the TPR for procedure that compute voxelwise statistical significance at a one‐sided significance level of α=0.05 without controlling for multiple comparisons. The TPRs (top row) and relative FPRs (bottom row) are shown for GDSS (green), FDR (violet), topological FDR (dark brown), TFCE (violet), and pTFCE (light brown). Top row: TPRs increase for increasing amounts of smoothness as decreasing noise increases statistical power to detect the added signal. As statistical power of the added signal increased, TPR for all statistical procedures increased from near zero to 100%. Note, however, that GDSS relative to all other procedures had greater TPR at lower statistical power, with TFCE having a slightly higher TPR than GDSS at higher statistical powers. FDR was most conservative and hence had the lowest TPR. When the added signal had sufficiently high statistical power, all procedures correctly rejected the null hypothesis (i.e., detected true positives) at all voxels with the added signal in the imaging volumes. Bottom row: FPRs increased for increasing amounts of smoothness likely because smoothing spread of the signal to voxels neighboring the region with the added signal. FDR had the lowest relative false positive rate, whereas pTFCE and TFCE had the highest FPRs for smoothness at FWHM = 2. At FWHM = 4, 7, and 10 voxels, GDSS had the highest FPRs because smoothing spread the signal, leading to the rejection of the null hypothesis at the neighboring voxels. The higher FPR for GDSS is attributable to spatial smoothing and therefore rejection of the null hypothesis at voxels that were spatially adjacent to the sphere with added signal. The FPRs for region of radius 10 voxels are much higher than region with radius 5 voxels, especially at FWHM = 7 and 10 voxels. That is, large amounts of smoothing can spuriously enhance the spatial extent of the findings in the data. FWER, familywise error rate; FWHM, full width at half maximum; FDR, false discovery rate; FPR, false positive rate; GDSS, geometry‐derived statistical significance; pTFCE, probabilistic TFCE; TFCE, threshold free cluster enhancement; TPR, true positive rate

Findings were similar to those for spherical region of radius 5 voxels: both TPR and FPR increased for increasing amounts of smoothness in the simulated data. TPR was higher for GDSS than other comparator procedures. TPR for all procedures was >99% when voxelwise statistical power was 0.189 in the unsmoothed data (Figure 7). TFCE and pTFCE had the highest FPR at FWHM = 2 voxels, whereas GDSS typically had similar or higher FPR than TFCE at FWHM = 4, 7, and 10 voxels. FPR increased with increasing amounts of smoothness largely because voxels neighboring the sphere were also deemed statistically significant due to image smoothing. Consequently, FPRs increase for all procedures with increasing strengths of signal and smoothness in the synthetic data (Figure 8). Finally, smoothing increased FPR more in data with region of radius 10 mm than in data with regions of radius 5 mm (Figures 6 and 8). FPR increased greatly when FWHM was increased from 4 to 7 voxels, thereby suggesting that a small increase in smoothness can profoundly affect the performance of the statistical procedures (Figure 8).

#### 3.2.3 Synthetic data without signal

The procedure that did not control for multiple comparisons and pTFCE rejected the null hypothesis at one or more voxels and therefore their FWER = 1 (Table 2). Furthermore, their FWER did not change for increasing amounts of smoothness in the simulated data. FWER for GDSS was close to 1 when imaging data were smoothed at FWHM = 4 voxels and decreased to 0.8 as FWHM was increased to 10 voxels (Table 2). These findings suggest that pTFCE and the proposed GDSS would almost always detect false positive findings in the data. Topological FDR had lower FWER than GDSS and pTFCE. TFCE and FDR had FWER was less than 0.06 for TFCE and FDR. High FWER for GDSS, pTFCE, and topological FDR suggests that these procedures as expected do not control for FWER in the strictest sense.

**TABLE 2:** We generated 250 sets of synthetic data on a 100 × 100 × 100 voxels in 3D space

	FWHM (voxels)	Uncorrected	Topological FDR	FDR	GDSS	TFCE	pTFCE
**FWER**	4	1	0.859	0.06	0.988	0.076	1
	7	1	0.46	0.048	0.892	0.072	1
	10	1	0.352	0.052	0.808	0.096	1
**Significant Voxels (Average)**	4	100,443	317	9	564	16	841
	7	100,539	553	48	1236	80	1183
	10	100,647	1090	94	2208	151	1133

For each of these 250 sets of synthetic data, we simulated data from 50 participants whose ages were selected at random from a Gaussian distribution with mean age of 20 years and standard deviation of 2 years. We therefore generated a total of 250 sets ×* 50 volumes/set = 12,500 volumes of synthetic data. Voxel intensity was independent of the age and was independently distributed according to the Gaussian distribution with mean of zero and variance of 1.0. Each of these 12,500 volumes were subsequently smoothed by applying a Gaussian kernel with full width at half maximum (FWHM) of either 4, 7, or 10 voxels. We then applied linear regression analysis at each voxel and used the five comparator procedures for ascertaining statistically significant associations. FWER was computed as the fraction of dataset in which the null hypothesis was rejected at one or more voxels. We also counted the number of significant voxels in each set and computed the average number across the 250 sets. The average number of significant voxels was rounded down to the closest integer.

FWER: equaled 1 for the uncorrected and pTFCE methods, decreased from almost 1 to 0.8 for the GDSS, decreased from 0.85 to 0.35 for topological FDR, and was less than 0.1 for both FDR and TFCE. Significant voxels: As expected, the number of voxels at which the null hypotheses were rejected was 100,000 in analysis that did not control for multiple hypotheses testing. Although FWER was close to 1 for GDSS, pTFCE, and topological FDR, the number of significant voxels was less than 2200 at FWHM = 10 voxels. These significant voxels were sparsely scattered as small 3‐voxel wide regions across the imaging volume.

Although the FWERs were high, null hypotheses were rejected at only a few voxels on average across the 250 datasets: null hypotheses were incorrectly rejected at fewer than 317 voxels for topological FDR, 841 voxels for pTFCE, and 564 voxels for GDSS procedures when the imaging data were smoothed at FWHM = 4 voxels (Table 2). The significant voxels increased for increasing amounts of smoothness because smoothness increased spatial correlations across neighboring voxels. Nevertheless, at an optimal FWHM = 4 voxels, null hypotheses were rejected at fewer than 0.0008 (<0.08%) of all voxels. These significant voxels were distributed sparsely as regions that were three voxels or less wide across the imaging volume.

### 3.3 Real‐world dataset

**FIGURE 9:**
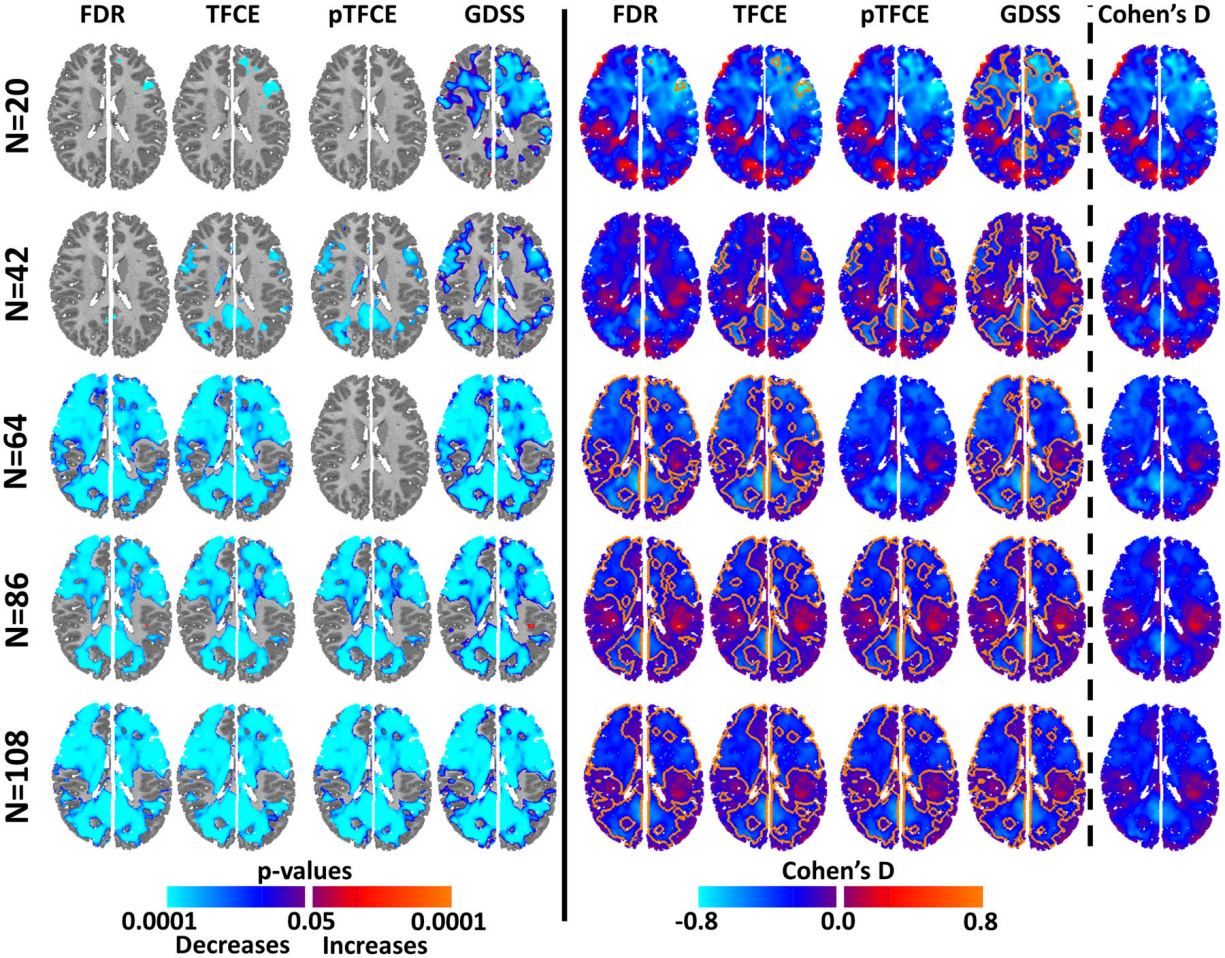
Age effects on blood perfusion in subsamples of participants subsamples were selected randomly from the entire cohort of 108 participants. Our null hypothesis was that regional cerebral blood flow was not associated with age, which we tested using multiple linear regression while covarying for sex, full scale IQ, and diagnosis. Statistical significance of the test statistic was ascertained using the various procedures for multiple comparisons correction. FDR was applied at an FDR = 0.05, and TFCE and pTFCE were applied to the data within SPM12. The *p*‐value map generated with pTFCE was threshed at probability −logP = 13.5 to detect statistically significant voxels, as none of the *p*‐values survived GRF‐based familywise error (FWER) correction. Left panel: The *p*‐value for voxels that survived correction for multiple comparisons was color coded and displayed on the template brain. Purple and blue: regions where perfusion decreases with age; red and orange: regions where perfusion increases with age. Regions that survived GDSS were spatially larger than those that survived FDR, TFCE, and pTFCE correction. Right panel: We computed maps for Cohen's D effect size and overlayed the boundaries (shown in orange lines) for regions in the left panel deemed to be statistically significant by each of the statistical procedures. These maps showed that for *N* = 20, GDSS (but not other procedures) rejected the null hypotheses at all voxels having a medium‐to‐large effect size. All procedures however correctly rejected the null hypothesis (detected all true positives) for *N* ≥64

The voxelwise measures of rCBF decreased significantly with age and survived the various procedures to control for multiple comparisons (Figure 9). For increasing numbers of participants, and hence for increasing statistical power at each voxel, rCBF was significantly associated with age across increasingly larger regions. For a small number of participants (*N* = 20), the statistically significant region was small for TFCE, FDR, and topological FDR, even though Cohen's D effect size was medium‐to‐large across large portions of the image (Figure 9, right panel). GDSS correctly rejected the null hypothesis across brain regions with medium‐to‐large effect sizes (Figure 9, left panel), thereby providing much greater statistical power than the other procedures for detecting true positives. For higher numbers of participants (*N* ≥64), all procedures identified similarly large regions as being statistically significant (Figure 9, left panel), even though Cohen's D effect size declines as the number of participants increased (Figure 9, right panel). Despite the decline in effect size, an increasing number of participants increased statistical power at each voxel, thereby permitting all statistical procedures to correctly reject null hypothesis. Associations did not survive pTFCE applied to subsamples with *N* = 20 or *N* = 64 participants when either GRF or a threshold of z‐score = 3.77 was applied to the probability maps. Overall, these findings show that GDSS provides the greatest statistical power to detect true positives while controlling for multiple comparisons.

**FIGURE 10:**
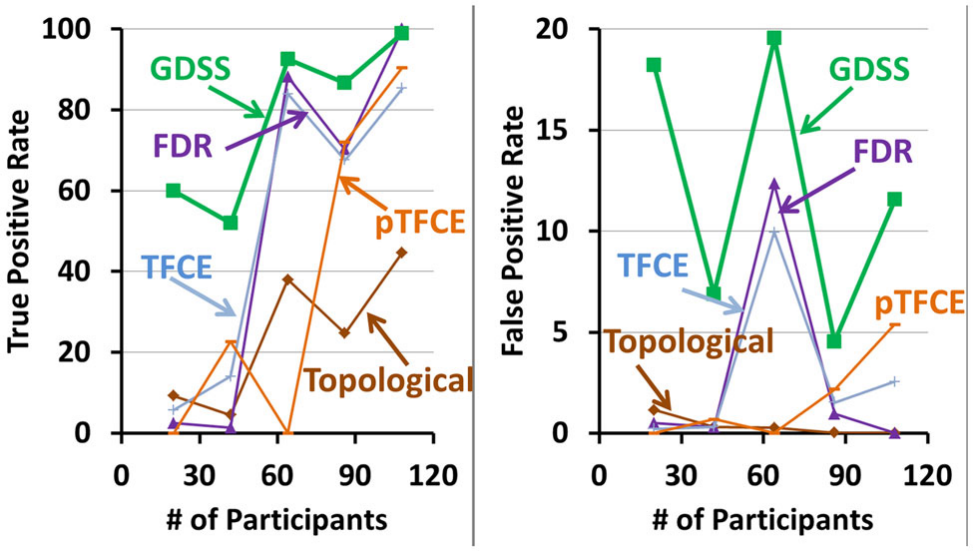
True positive rate (TPR) and false positive rate (FPR) for increasing number of participants. We assumed that associations that survived the false discovery rate (FDR) procedure applied to the entire cohort of 108 participants represents true associations in the data. Although the true associations between age and regional cerebral blood flow may differ across subsamples with different participants, findings that survived the FDR procedures permit comparison of the performance of the various statistical procedures. For each statistical procedure and for increasing number of participants, we computed TPR and FPR and plotted them for increasing number of participants. Green curve: the proposed GDSS procedure; dark brown curve: the topological FDR procedure; violet curve: the FDR procedure; light blue curve: the TFCE procedure; and light brown curve: the pTFCE procedure. Left: The TPR for the GDSS procedure is significantly higher than the other procedures across all subsamples, especially when the number of participants is small. The TPR was lowest for the topological FDR procedure. Right: The FPR for the proposed GDSS procedure was high for all subsamples. GDSS ascribes significance to contiguous regions of voxels and, therefore, the high FPR likely is due to greater spatial extent of the significant regions than that for those for the significant regions that survived the FDR procedure (Figure 9)

We computed TPRs and FPRs for all statistical procedures relative to findings generated with FDR applied to the entire cohort of 108 participants (Figure 10). GDSS had the highest TPR, especially when the number of participants was <64 and statistical power at each voxel was low (Figure 10, left panel). Although the true findings in the subsample of 20 participants may differ somewhat from true findings in the entire cohort of 108 participants, the voxelwise map for Cohen's D effect size (Figure 9, right panel) showed GDSS ascribed statistical significance only to voxels with medium‐to‐large effect sizes, and hence it correctly rejected the null hypothesis (detected true positives) at those voxels. The FPR for GDSS was also higher than for all other statistical procedures (Figure 10, right panel). The higher FPR for GDSS likely was not due to ascribing statistical significance to differing set of regions, but rather to ascribing significance across spatially larger regions than the regions considered significant by FDR. Moreover, the FPR for GDSS was high (15%) across all subsamples, possibly because FDR had a lower statistical power to detect true findings, and hence, FDR likely failed to reject the null hypothesis.

**FIGURE 11:**
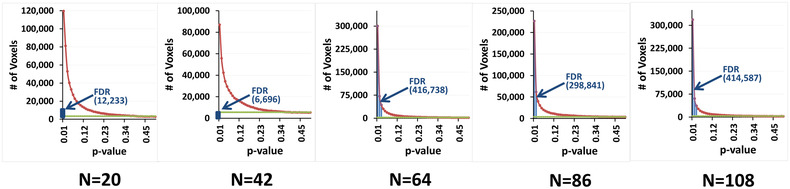
Distributions of *p*‐values for the associations of regional cerebral blood flow with age in real‐world data. We computed one‐sided *p*‐values, as our a priori alternate hypothesis was that regional blood flow declines with age. The distribution was plotted as a histogram with bins of size 0.01. The *x*‐axis represents *p*‐values, the *y*‐axis represents the number of voxels having a *p*‐value within a specified bin. The dark red curve shows the distribution of the *p*‐values; the light green line shows the uniform distribution for *p*‐values if the statistic was distributed according to the null hypothesis; blue bars at the left show the *p*‐values that were rejected, and hence that were deemed statistically significant, by FDR. The dark red curve shows that the *p*‐value distribution deviated significantly from the uniform distribution (light green line) for small *p*‐values. FDR cannot discriminate *p*‐values for the null from those for the alternate distribution and hence rejects all small *p*‐values up to a certain value computed empirically from the data. When data are available for only a limited number of participants (*N* ≤ 42), statistical power is low and hence the null and the alternate distributions overlap substantially. FDR, however, rejected only a small portion (12,233 for *N* = 20 and 6696 for *N* = 42) of the smallest p‐values and failed to reject large numbers of *p*‐values that deviated from the uniform distribution. GDSS uses the local geometry of a random field to compute the probability for rejecting the null hypothesis. When the number of participants was large (>64 in this dataset), the statistical power to reject the null hypothesis at each voxel was large, which is apparent from the sharp and narrow peak of p‐values close to 0. At high statistical power, FDR correctly rejected large numbers of null hypotheses (416,738 for *N* = 64; 298,841 for *N* = 86; 414,587 for *N* = 108) and yielded findings that matched closely to those for GDSS (Figure 9)

The distributions of *p*‐values (Figure 11, dark red curve) computed under the theoretical null hypothesis deviated from a uniform distribution (Figure 11, light green line), suggesting that the test statistic in a large portion of the brain were distributed according to the alternate hypothesis. Because FDR cannot discriminate between *p*‐values for the test statistic under the null hypothesis from those under the alternate hypothesis, FDR rejects all *p*‐values smaller than an empirically estimated value (Figure 11, blue bars). Figure 9 shows that FDR failed to reject a large proportion of *p*‐values that likely were from the alternate hypothesis (i.e., it generated many false negative findings). For example, in the *N* = 20 subsample, FDR rejected the null hypothesis only on 12,233 voxels, even though *p*‐values deviated from the uniform distribution at hundred thousands of voxels. In the *N* = 42 subsample, even fewer voxels (6696) survived FDR correction, suggesting that FDR failed to reject the null hypothesis at hundred thousands of voxels and therefore had very low statistical power to detect true positives. GDSS uses a geometry‐derived probability to reject the null hypothesis and thereby greatly improves statistical power compared to FDR.

**FIGURE 12:**
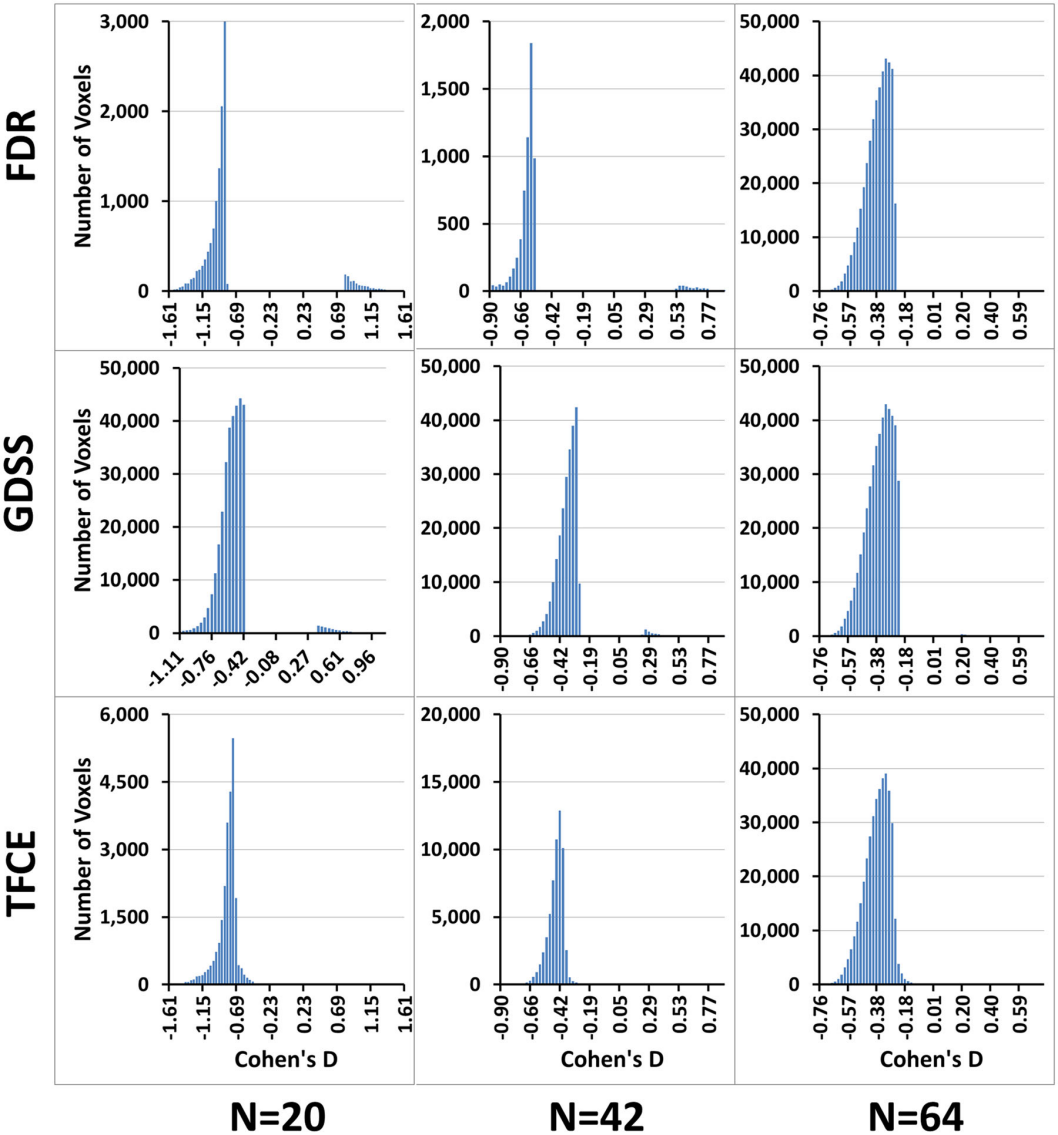
Distribution of the Cohen's D effect size histograms are shown for voxels where the null hypothesis was rejected by FDR, GDSS, and TFCE for the 20, 42, or 64 participant subsamples. Similar distributions for the 86 and 108 participant subsamples are not shown. These histograms show that for the 20 or 42 participant subsamples, FDR rejected the null hypothesis at fewer voxels with a large Cohen's D effect size than did GDSS. Although TFCE rejected the null hypothesis even for very small effect sizes, it rejected the null at far fewer voxels than GDSS, especially for the *N* = 20 and *N* = 42 subsamples. Thus, GDSS provided significantly greater statistical power to detect true positives at voxels with medium‐to‐large effect sizes, and correctly did not reject the null hypothesis when the effect size was small

We generated histograms for Cohen's D effect size at voxels ascertained to be statistically significant (Figure 12). These histograms show that GDSS identified a larger number of voxels with medium‐to‐large effect sizes as being statistically significant than the other procedures evaluated. FDR and TFCE failed to reject the null hypothesis (they yielded false negatives) even at voxels with medium‐to‐large effects.

**FIGURE 13:**
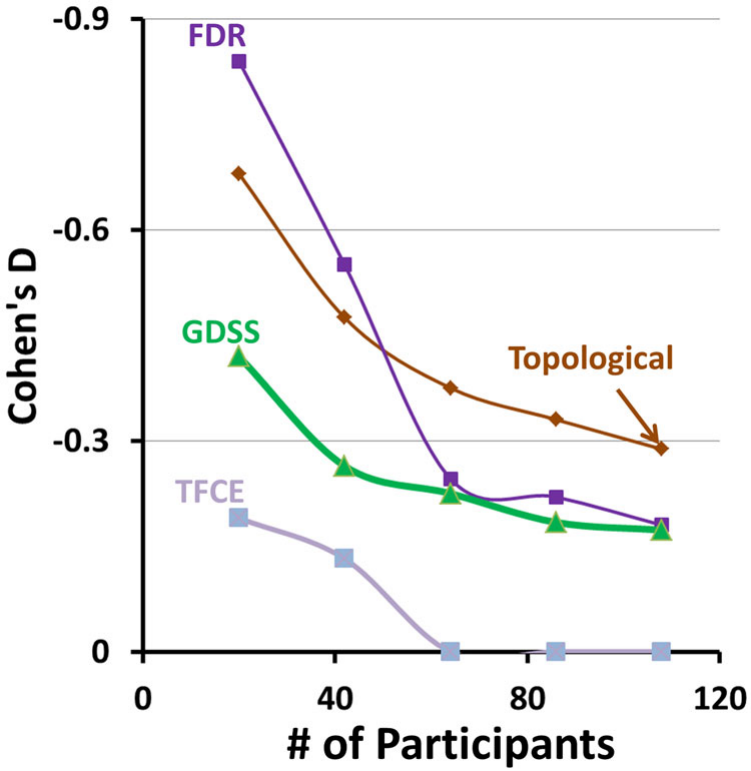
The smallest Cohen's D effect size rejected by each statistical procedure. We plotted the value of the smallest Cohen's D effect size for voxels where the null hypothesis was rejected against the number of participants in each subsample. Violet curve: FDR; dark brown curve: topological FDR; dark green curve: GDSS; light blue curve: TFCE. Because FDR requires sufficiently large statistical power at each voxel, the smallest Cohen's D effect size for which FDR rejects the null hypothesis is large for 20 or 42 participants. However, with 64 or more participants, the smallest Cohen's D effect size for FDR is similar to GDSS. For TFCE, the smallest Cohen's D effects size is lowest and reaches 0 for 64 and higher participants. These findings suggest that TFCE was least conservative among these procedures in rejecting the null hypothesis, though its TPR was lower than for GDSS ( Figure 10)

We plotted the smallest Cohen's D effect size at voxels where the null hypothesis was rejected (Figure 13). FDR was the most conservative for the *N* < 64 subsamples because FDR requires sufficiently high statistical power at each voxel (i.e., sufficiently small *p*‐values) to rejecting the null hypothesis. Voxelwise statistical power increased with the number of subsample participants, and hence the smallest Cohen's d effect size decreased for the FDR procedure (Figure 13). The proposed GDSS procedure rejected the null hypothesis at voxels with smaller Cohen's D effect sizes, especially in subcohorts with *N* < 64 participants, suggesting that the GDSS procedure will have much higher statistical power than the FDR procedure. Comparatively, the TFCE procedure rejected the null hypothesis at voxels with very small effect size: for subcohorts with N≥64, the null hypothesis was rejected at voxels even with Cohen's D effect size of 0, thereby suggesting that the null hypothesis was rejected even where there was no association between the “age” and rCBF. That is, the TFCE procedure was the least stringent in rejecting the null hypothesis.

**FIGURE 14:**
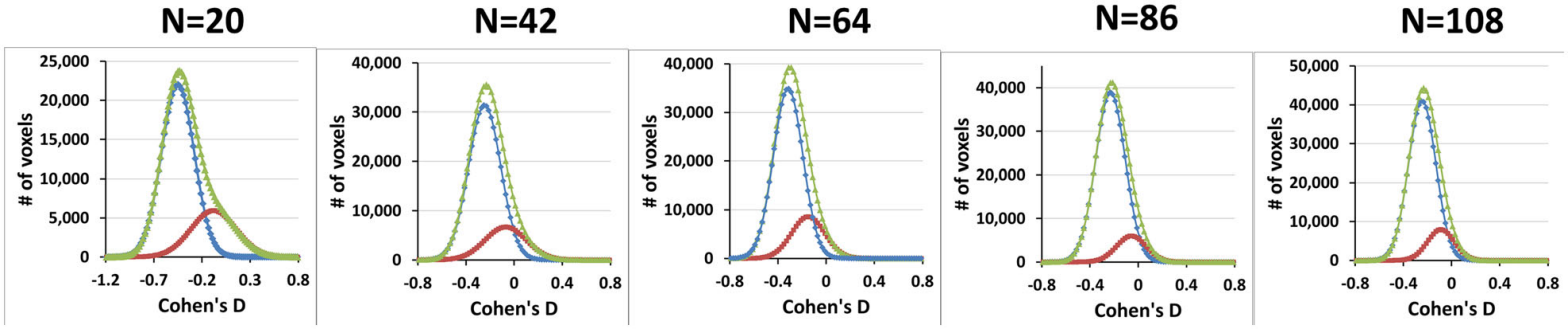
Modeling distributions of Cohen's D effect size as a weighted sums of two Gaussian density functions. From the map of test statistics across the brain, we computed Cohen's D effect size at each voxel and then generated its distribution as a histogram. Green curve: the distribution of effect sizes across the brain; blue and red curves are the two distinct Gaussian functions whose weighted sum equals the green curve. The distribution of effect sizes is accurately represented as a weighted sum of two Gaussian distributions: the blue curve represents the distribution of the effects sizes under the alternate hypothesis, and the red curve denotes the distribution under the null hypothesis. Under the null hypothesis, the effect size distribution was centered close to zero (from 0.04 to −0.08),whereas under the alternate hypothesis, the distribution was centered at decreasing effect sizes, from 0.45 to 0.23, as the number of participants increased from 20 to 108

**FIGURE 15:**
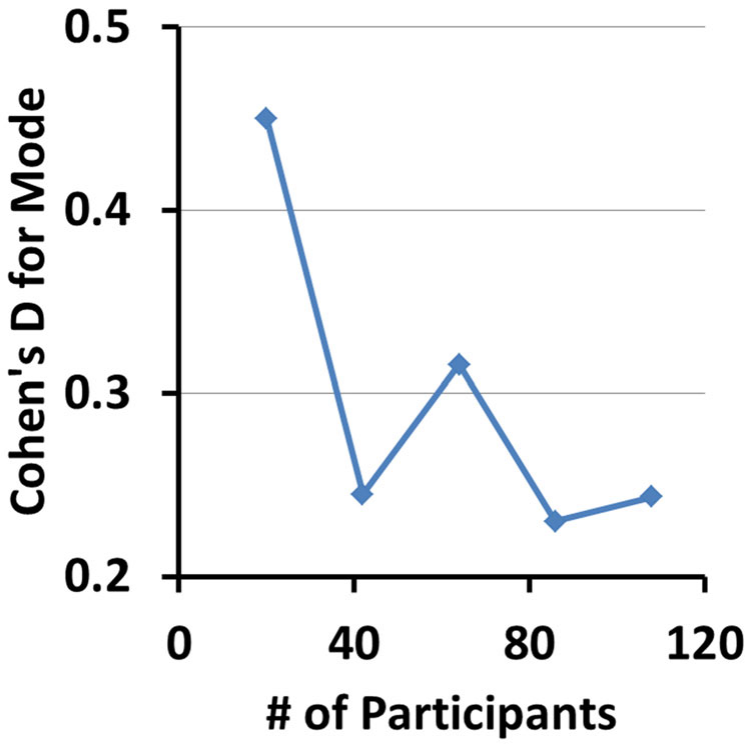
Cohen's D effect size declined with the number of participants. We plotted the mean effect size for the Gaussian distribution under the alternate hypothesis. The absolute value of the effect size was 0.45 for the 20 participant subsample, which decreased to 0.25 as the number of participants increased to 108. The nonsmooth decline in mean effect size likely was due to the random selection of participants in subsamples from the entire cohort of 108 participants. Furthermore, the effect size decreased for increasing numbers of participants because of the averaging of the effect across participants with heterogeneous demographic and clinical characteristics. The decline in effect size with increasing numbers of participants raises concerns about using effect sizes from small cohorts for sample size calculations planned for larger, more definitive studies. A larger number of participants is more likely needed than the numbers computed using estimated effect sizes obtained in small pilot studies

We generated the distribution of Cohen's D effect size across the entire brain and fitted a weighted sum of two Gaussian distributions, one modeling the distribution for the alternate hypothesis and the other modeling the distribution for the null hypothesis (Figure 14). The Gaussian distribution with mode centered near 0 likely represented the distribution for the null hypothesis, whereas the other Gaussian distribution, with its mode at −0.45 to −0.24, likely represented the distributions for the alternate hypothesis (Figure 14). The fitted Gaussian distributions revealed that Cohen's D effect size declined as the number of participants increased due to averaging of the effect across increasing numbers of demographically and clinically heterogeneous participants (Figure 15). To test this possibility, we generated a new set of 20 different subsamples, each with 20 participants sampled randomly from the 108 participants. In each of the 20 subsamples, we tested the null hypothesis across the entire brain, computed and formed the distribution for Cohen's D effect size, and fitted the weighted sums of two Gaussian distributions for each. We then found the mean and standard deviation for the mode of these 20 distributions to be −0.36 and 0.119, respectively, which differed significantly from the mode of −0.24 for the entire cohort of 108 participants (Student's *t* = 4.69, df = 19, two‐sided *p* = 0.0002). These findings provide strong support for the assertion that effect sizes decline with an increasing number of participants.

**FIGURE 16:**
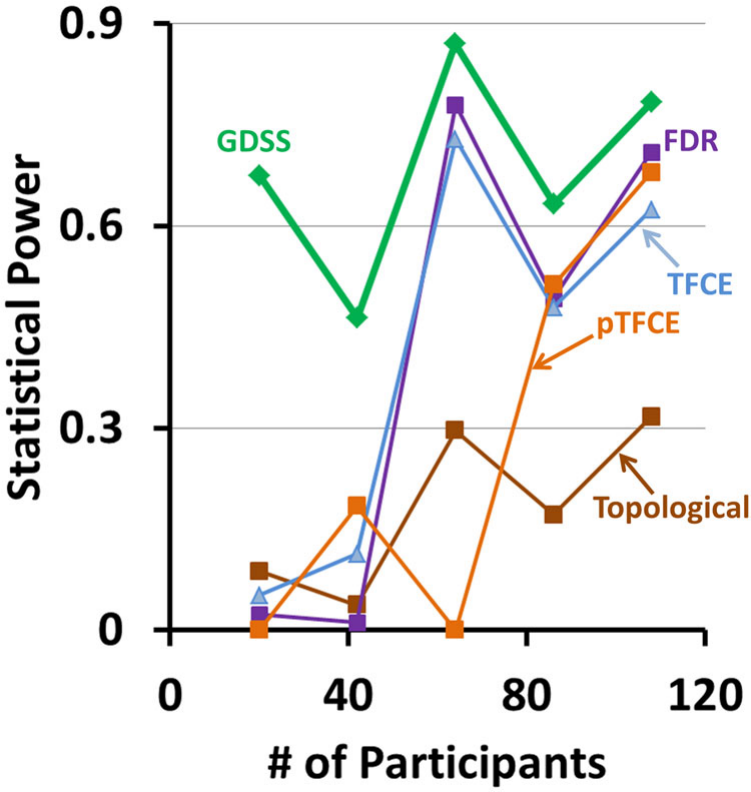
Estimating the statistical power for the various statistical procedure. We fitted a weighted sum of two Gaussian distributions to the distribution of Cohen's D effect sizes, which permitted us to estimate the number of voxels that had the test statistic distributed according to the alternate hypothesis. Using these estimated number of voxels and the number of voxels at which the null hypothesis was rejected, we computed the fraction of voxels (i.e., the statistical power) at which the null hypothesis was correctly rejected when in fact the alternate hypothesis was true. We plotted the statistical power for increasing number of participants for each of the statistical procedure. Green curve: statistical power for the proposed GDSS procedure; dark brown curve: statistical power for the topological FDR procedure; violet curve: statistical power for the FDR procedure; light blue curve: statistical power for the TFCE procedure; light brown curve: statistical power for the pTFCE procedure. The proposed GDSS procedure had the highest statistical power, which was largely invariant to the number of participants. The statistical power for other procedures was close to 0 for subsamples of up to 42 participants, and although their statistical power increased for more than 42 participants, it remained lower than that for the GDSS procedure

Using the fitted Gaussian distribution for the alternate hypothesis (Figure 14) and the voxels where the null hypothesis was rejected (Figure 12), we computed the statistical power that each procedure provided in each of the subsamples (Figure 16). These plots showed that statistical power for GDSS was 0.6 or higher across all subsamples. In contrast, statistical power for the comparison procedures was 0.1 or lower for 42 or fewer participants, and it was lower than for GDSS even samples with 64 or more participants. Thus, GDSS provided the greatest statistical power to detect true positive findings.

**FIGURE 17:**
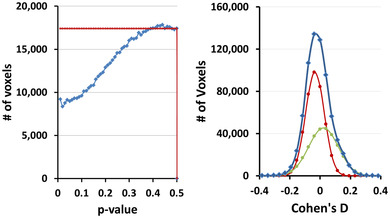
Age‐by‐diagnosis effects on regional cerebral blood perfusion in our cohort of 43 participants with Autism Spectrum Disorder (ASD) and 65 typically developing (TD) individuals. We applied a multiple linear regression model $y_{i}=\beta_{0}\;+\beta_{1}*age+\beta_{2}*sex+\beta_{3}*FSIQ+\beta_{4}*Dx+\beta_{5}*age*Dx+\varepsilon_{i}$, where $y_{i}$ is the regional cerebral blood flow (rCBF) value at a voxel across each of the 108 participants. The uncorrected, voxelwise analyses showed rCBF in a few small brain regions was significantly associated with age‐by‐diagnosis effects; that is, age effects on rCBF differed by diagnosis. However, these small regions did not survive the control for multiple hypotheses testing by the proposed GDSS procedure. To understand why the age‐by‐dx effects were not statistically significant, we plotted the histogram of *p*‐values and the histogram of Cohen's D effect sizes. Left *:* The histogram for *p*‐values (blue curve) deviated from the theoretical uniform distribution (red line) and that greater number of voxels have *p*‐values close to 0.5. The deviation from uniform values suggests that the test statistics were derived from two distinct distributions rather than the theoretically assumed Gaussian distribution. Furthermore, greater numbers of *p*‐values near 0.5 than near 0.0 suggest that the two Gaussian distributions were centered at the null effect. Right *:* We computed Cohen's D effect size from Student's *t*‐test statistic at each voxel in the brain and plotted its distribution (dark blue curve). We subsequently fitted a sum of two weighted Gaussian distributions (red and green curves). As we expected from the distribution of the *p*‐values (left), the modes (−0.034 and 0.0241) for the two distributions were centered close to zero. These two distributions likely represent the distributions of test statistic under the null and the alternate hypotheses

Our prior analyses had shown that the age effects in ASD participants largely did not differ from those in TD participants; that is, age‐by‐diagnosis effects on rCBF were present only at small regions in the brain. Some of these uncorrected voxels also survived the FDR procedure to control for multiple hypothesis testing. However, the null hypothesis at these voxels was rejected by the GDSS, TFCE, or pTFCE procedures. To understand why the GDSS procedure failed to reject the null hypothesis, we plotted the histogram of *p*‐values and also the uniform distribution under the null hypothesis (Figure 17, left panel). The histogram showed that there were fewer voxels for *p*‐values < 0.3 than would be expected from the average number of for *p*‐values > 0.35 in a uniform distribution; and therefore, the null hypothesis was not rejected at any voxel by the GDSS procedure. Furthermore, greater numbers of voxels had *p*‐values close to 0.5 than the number of voxels with *p*‐values close 0.0, suggesting that test statistic are distributed from two distinct density functions, both of which are centered close to mean of 0.0. We therefore computed and plotted the histogram for Cohen's D effect sizes and fitted a weighted sum of two Gaussian distributions (Figure 17, right panel). As we had expected, the two Gaussian distributions were centered close to mean of zero. Thus, although a few isolated voxels were ascertained to be statistically significant by the FDR procedure, the proposed GDSS procedure likely correctly failed to reject the null hypothesis.

## 4 DISCUSSION

GDSS is a new statistical procedure that corrects *p*‐values for the multiple statistical comparisons inherent when testing hypotheses in contemporary MRI datasets. GDSS combines *p*‐values with the probabilities that derive from the local geometry of a random field under the null hypothesis. It does not require specifying a CDT, but instead defines contiguous regional findings by maximizing a posterior probability through use of a Gibbs distribution as a prior on region definitions. Similar to FDR, GDSS is based on the fact that *p*‐values under the null hypothesis have a uniform distribution and the corresponding assumption that *p*‐values which deviate from the uniform distribution derive from the alternate hypothesis. Because FDR cannot discriminate *p*‐values for the null from those for the alternate hypothesis, it rejects all *p*‐values smaller than a value that is estimated empirically from the data. We contend that local geometry of test statistic in the neighborhood of a voxel can be used to compute geometry‐derived probability for the test statistic under the null hypothesis that brain measure is not associated with outcome measure. Using probabilities for local geometry, GDSS discriminates *p*‐values under the null from those under the alternate hypothesis. This ability to discriminate and reject the null hypothesis provided significantly greater statistical power to reject the null hypothesis.

We evaluated the performance of GDSS and compared it with the performance of the most commonly employed procedures applied in the MR imaging analyses: FDR, topological FDR, TFCE, and pTFCE. We assessed performance using synthetic data containing regions with known signal for increasing amounts of smoothness and real‐world data containing robust associations of rCBF with age. GDSS provided greater statistical power to detect true positive findings, especially when statistical power to discriminate the null from alternate hypothesis at each voxel was low (Figures 3, 4, 5, 6, 7, 8). GDSS had lower FPRs when the data were smoothed by small amounts using FWHM = 2 voxels, but had higher FPR when data were smoothed by very large smoothing kernels with FWHM = 7 and 10 voxels. However, at FWHM = 7 and 10 voxels, FPR was much higher for all statistical procedures. Our simulated data suggests that a use of smoothing kernel with FWHM = 4 voxels provided optimal balance between true and FPRs for all statistical procedures. At this optimal FWHM, the FPR for GDSS was small and comparable to those for comparison procedures, thereby suggesting that GDSS rejected null hypotheses within region whose spatial extent did not differ substantially from the region with added signal. GDSS afforded more statistical power by detecting more true positives than the comparison procedures, even though it was more conservative by permitting fewer false positives than both TFCE and pTFCE in the reported findings: their FPRs were higher in smaller clusters with greater effect sizes (Figures 4, 5, 6). Synthetic data without any added signal showed that GDSS, pTFCE, and topological FDR procedures almost always rejected the null hypothesis at a few voxels when the imaging space is sufficiently large (Table 2). However, voxels at which the null hypothesis was incorrectly rejected were sparsely distributed as small regions that were only three voxels wide. Very small regions with significant findings typically are considered noise and not interpreted or presented as findings in real‐world studies, and therefore, these statistical procedures can be considered to control for FWER in a weak sense. GDSS provided greater statistical power than the comparison procedures to detect true positives in real‐world data, particularly when the number of participants was small (Figures 9 and 10).

Parametric methods for statistical inference compute *p*‐values for a test statistic by assuming a specific theoretical distribution—typically a central Student's *t*‐distribution—under the null hypothesis. Brain imaging measures typically are Gaussian distributed because (a) brain measures vary smoothly across space, and (b) imaging data are smoothed using a Gaussian kernel with FWHM = 6 to 8 ms to reduce noise and when interpolated during registration into a template space. If the true empirical distribution differs from the assumed theoretical one, then the *p*‐values computed for the test statistic will not have a uniform distribution, even in the absence of statistically significant effects. These deviations of the empirical from theoretical distribution will greatly influence the accuracy of not only GDSS but also FDR and other statistical procedures that use GRF‐based methods for FWER correction. These errors in inference may be avoided by employing nonparametric statistics, such as the permutation approaches that TFCE employs. Our real‐world data, however, suggests that the distributions for effect sizes and test statistics can be approximated as weighted sums of two Gaussian distributions, one corresponding to the null hypothesis and the other to the alternate hypothesis.

One may not be able to discriminate whether the test statistic is distributed as a bimodal distribution under the null hypothesis or as a sum of a null and an alternate hypothesis, especially when the effect size is small. For example, the distribution of the age‐by‐diagnosis effect on the rCBF was best described by a weighted sum of two Gaussian distributions, where both distributions had mean close to zero. This bimodal distribution could either be (1) a single, true empirical distribution under the null hypothesis, or (2) a sum of a unimodal distribution under the null hypothesis and another unimodal distribution under the alternate hypothesis, with the mean of the two distributions close to zero. Whether the null hypothesis is unimodal or bimodal, however, can be discriminated only if the number of participants is increased: if the true distribution is bimodal under the null hypothesis, then it will remain bimodal for increasing number of participants. In contrast, if the bimodal distribution represents a sum of two distributions under the null and the alternate hypotheses, then increasing the number of participants would decrease the standard error and increase the statistical power to discriminate test statistics from the two distributions. For the interaction effect of age‐by‐diagnosis on rCBF, the effect size was small, and therefore, the use of theoretical unimodal distribution computed *p*‐values close to 0.5 were from both the null and the alternate hypotheses. This caused an overestimation of the number of voxels from the null hypothesis, and consequently, GDSS did not reject the null hypothesis and possibly leading to false negatives relative to FDR.

Our findings also showed that the voxelwise Cohen's D effect size declines with an increasing number of participants, likely because of the averaging of effects across heterogeneous participant subsamples. Cohen's D in 20 participant subsample was substantially higher than in the sample of all 108 study participants (Figure 15). This decrease in effect size with increase numbers of participants suggests that estimating effects in larger samples from small samples of clinically heterogeneous participants may be invalid. It also suggests that using effect sizes from a small pilot study to calculate sample size for a larger study is hazardous and likely to be an underestimate.

Finally, our findings suggest that imaging studies should report not only *p*‐values at voxels deemed statistically significant but also the associated Cohen's D effect sizes to interpret the findings correctly. *p*‐value maps present only a partial picture of the data and do not indicate whether statistical significance was attributable to a large effect size or a large number of study participants. *p*‐value maps also fail to indicate whether the entire homogeneous region with medium‐to‐large effect size or only a portion of it has been deemed to be statistically significant. Therefore, several researchers in the past have suggested reporting of effect sizes and confidence intervals to truly assess the significance of the reported findings (Gelman & Geurts, 2017; Gelman & Loken, 2014; Kelley & Preacher, 2012; Schmidt, 1996). Our findings show, for example, that FDR and TFCE in general deem only small portions of regions with large effects to be statistically significant. GDSS, in contrast, deems much larger portions of regions with medium‐to‐large effect sizes to be statistically significant.

## 5 CONCLUSIONS

Our findings show that the proposed GDSS procedures provides greater statistical power to detect true positives than procedures that are currently employed in the field of MRI, especially in datasets with fewer than 40 participants. Although the proposed methods continued to have greater statistical power, other procedures including FDR and TFCE had comparable statistical power for larger cohorts. The higher statistical power possibly came at a cost of greater FPRs in GDSS relative to FDR, and hence, GDSS might be employed when maximization of TPR is prioritized over minimizing FPR in a study. Topological FDR provides improvement over FDR for both TPR and FPR relative to FDR. TFCE and pTFCE overcome the limitation of the need for specifying CDT and provide much higher TPR than topological FDR. Nevertheless, TFCE can be least stringent by permitting voxels with small Cohen's D effect size deemed as being statistically significant. In contrast, GDSS provided consistently high statistical power across differing size datasets and only voxels with medium to large effect sizes were deemed as statistically significant. GDSS however is limited by the distribution of *p*‐values that are computed for an assumed theoretical distribution for test statistic. If the empirical distribution differs from the theoretical distribution, then the test statistics from the null hypothesis could be deemed as statistically significant by GDSS. For example, if the theoretical null distribution is assumed to be a Gaussian distribution but the empirical distribution is a bimodal distribution, then the histogram for *p*‐values will deviate away from a uniform distribution and null hypothesis will be rejected even though test statistic are distributed according to the null hypothesis. Therefore, the correct use of GDSS requires accurate specifications of the distribution under the null hypothesis. To summarize, GDSS can be employed when minimization of false negatives is prioritized over minimization of false positives when conducting multiple hypotheses testing across brain imaging data.

## CONFLICT OF INTEREST

All authors certify that they do not have any affiliations with or involvement in any organization or entity with any financial interest (such as honoraria; educational grants; participation in speakers’ bureaus; membership, employment, consultancies, stock ownership, or other equity interest; and expert testimony or patent‐licensing arrangements), or non‐financial interest (such as personal or professional relationships, affiliations, knowledge or beliefs) in the subject matter or materials discussed in this manuscript.

### PEER REVIEW

The peer review history for this article is available at https://publons.com/publon/10.1002/brb3.2865.

## Data Availability

All source code and data will be made available upon request for research purposes.
